# Synthesis of Terpolymers with Homogeneous Composition by Free Radical Copolymerization of Maleic Anhydride, Perfluorooctyl and Butyl or Dodecyl Methacrylates: Application of the Continuous Flow Monomer Addition Technique

**DOI:** 10.3390/polym9110610

**Published:** 2017-11-13

**Authors:** Marian Szkudlarek, Uwe Beginn, Helmut Keul, Martin Möller

**Affiliations:** 1 DWI Leibnitz Institute for Interactive Materials and Institute of Technical and Macromolecular Chemistry, RWTH Aachen University, Forckenbeckstraße 50, D-52056 Aachen, Germany; marian.szkudlarek@ciechgroup.com (M.S.); keul@dwi.rwth-aachen.de (H.K.); 2 Institut für Chemie, OMC, Universität Osnabrück, Barbarastraße 7, D-49076 Osnabruck, Germany

**Keywords:** terpolymer, radical copolymerization, terpolymerization, homogenous composition, continuous monomer addition

## Abstract

Terpolymers of homogeneous composition were prepared by free radical copolymerization of butyl or dodecyl methacrylate, 1*H*,1*H*,2*H*,2*H*-perfluorodecyl methacrylate and maleic anhydride using the continuous monomer addition technique. The copolymerization reactions were performed at 65 °C in the presence of azobisisobutyronitrile as an initiator in a mixture of methyl ethyl ketone and 1,3-bis (trifluoromethyl)benzene. The monomers and initiator are added to the reaction mixture with the same rate they are consumed in 5- and 10-fold excess compared to the initial monomer stock. The obtained terpolymers with molecular weights *M*_n_ = 50,000–70,000 are of uniform composition, close to the composition determined in low conversion experiments, proving the principle of the chosen concept. The kinetic data necessary for the design of the continuous addition experiment were obtained from binary copolymerization experiments at low monomer conversion (to avoid compositional drift). In addition, the so-called terpolymerization parameter was determined from ternary copolymerization experiments.

## 1. Introduction

Due to their outstanding chemical resistance, thermal stability as well as low friction coefficient and flame retardation [1], fluorinated and partly fluorinated polymers have found many industrial applications, e.g., as friction modifiers [2], anti-fouling coatings [3] or membranes [4,5]. Homopolymerization of fluorinated monomers such as perfluoroolefins, (meth) acrylates with perfluorinated chains or fluorinated styrene derivatives leads to polymers with high fluorine content. The properties of copolymers obtained by employing these monomers might not always be optimal for specific applications. The use of fluorine containing monomers in polymer synthesis is under strong pressure from public organizations pointing out the adverse health effects such as enamel fluorosis due to excessive fluorine exposure [6]. The combination of the need to fine tune the polymer properties and limit the amount of the fluorine compound to the necessary minimum concentration (to still maintain all the benefits of using fluorinated materials) leads to the development of materials that are copolymers which contain highly fluorinated monomers reacted with non-fluorinated constituents. The copolymerization of usually very expensive fluorinated monomers with non-fluorinated monomers has also an economic effect of creating more cost effective materials that can be disposed easier, cheaper and with less impact on people and the planet. Batch free radical polymerization in binary or ternary systems usually leads to products which are blends of polymer chains with different composition. The alteration of the composition during the reaction is caused by different reactivity of the monomers in the mixture. Hence, the most reactive monomers are consumed first, and consequently the polymer is enriched in monomers of lower reactivity. This effect is strongly pronounced when monomers which cannot undergo homopolymerization are used. The preferred route to overcome this problem is to feed continuously the reaction mixture with monomers at the rate they are consumed. The present report focuses on the copolymerization of maleic anhydride with alkyl-methacrylates and 1*H*,1*H*,2*H*,2*H*-perfluorodecyl methacrylate. To ensure a homogenous composition of the terpolymers, continuous addition of the monomers was applied. The rate of addition was adjusted by the kinetics of the copolymerization. The composition of synthesized copolymers was determined by means of ^1^H NMR (nuclear magnetic resonance) spectroscopy and elemental analysis. The obtained copolymers were characterized by means of GPC, while the thermal behavior of the copolymers was investigated by means of TGA (thermal gravimetric analyzer) and DSC (differential scanning calorimetry).

## 2. Experimental

### 2.1. Materials

1*H*,1*H*,2*H*,2*H*-perfluorodecyl methacrylate (F8H2MA, Aldrich, St. Louis, MO, USA), *n*-butyl methacrylate (BMA, Merck, Kenilworth, NJ, USA) and dodecyl methacrylate (DMA, Merck) were distilled over CaH_2_ (Sigma-Aldrich Chemie, Steinheim, Germany) under reduced pressure. Maleic anhydride (MAH, Merck, for synthesis) was sublimed under reduced pressure (50 °C, 2.4 × 10^−2^ bar) before use. Azobisisobutyronitrile (AIBN, Merck) was recrystallized from methanol at 40 °C. 2-Butanone (MEK, Merck) was dried over CaH_2_ and distilled before use. 1,3-Bis (trifluorome-thyl)benzene (HFX, ABCR, Karlsruhe, Germany), Freon 113 (Fluka) and other solvents were used as received.

### 2.2. Measurements/Apparatus

Size exclusion chromatography (SEC) was performed with a system consisting of a LC 1120 pump (Polymer Laboratories, Church Stretton, UK), a UV detector ERC-7215 and RI detector ERC-7515A (ERMA CR INC, Kawaguchi City, Japan), a precolumn 50 × 8 mm with 50 Å nominal pore size and four columns (300 × 8 mm) filled with MZ-Gel SDplus of nominal pore sizes 50 Å, 100 Å, 1000 Å, and 10,000 Å (MZ-Analysentechnik, Mainz, Germany). The set was calibrated with PMMA (polymethyl methacrylate) and PS (polystyrene) standards from Polymer Laboratories. The sample concentration was 7 mg of polymer in 1 mL of solvent; the injected sample volume was 100 μL. Tetrahydrofuran (THF) used for measurements, and was stabilized with 2,6-di-*tert*-butyl-4-methylphenol (250 mg/L). Samples of high fluorine content were dissolved and measured in a mixture of THF: Freon 113 1:1 (vol:vol), stabilized with 250 mg/L of 2,6-di-*tert*-butyl-4-methylphenol.

^1^H NMR spectra were obtained on a Bruker DPX-300 (Bruker Corporation, Karlsruhe, Germany) spectrometer in CDCl_3_, acetone-*d*_6_ and mixtures with Freon 113 (1:1, vol:vol) at 300 MHz. MestRe-C 4.9.0.0 (Mestrelab Research, S.L., Santiago de Compostela, Spain) was used as evaluation software. Solvent signals were used as internal references. In the spectra description the maximum of the signal is given.

Thermogravimetric analysis (TGA) was performed using a Netzsch TG 209c (Netzsch, Selb, Germany) thermo balance under nitrogen atmosphere at a nitrogen flow 15 mL/min. Samples of 9–11 mg were placed in standard Netzsch alumina 85 μL crucibles and heated at a rate of 10 K∙min^−1^.

Differential scanning calorimetry (DSC) measurements were performed using a Netzsch DSC 204 unit. Samples (typical weight: ~9 mg) were enclosed in standard Netzsch 25 μL aluminium crucibles. Indium and palmitic acid were used as calibration standards. Heating and cooling rates were 10 K∙min^−1^.

Continuous monomer addition was performed with a Harvard Apparatus 11 Plus (Harvard Apparatus, Holliston, MA, USA) syringe pump equipped with 50 mL Braun syringes.

Elemental analyses were performed by Anastasya Buyanowskaya at the A.N. Nesmeyanov Institute of Organoelement Compounds of the Russian Academy of Science in Moscow.

### 2.3. Low Conversion Experiments

#### 2.3.1. Maleic Anhydride and 1*H*,1*H*,2*H*,2*H*-Perfluorodecyl Methacrylate

Maleic anhydride (0.65 g, 6.63 mmol) and 1*H*,1*H*,2*H*,2*H*-perfluorodecyl methacrylate (2.34 g, 4.7 mmol) were dissolved in a mixture of 2-butanone (2.25 g) and 1,3-bis (trifluoromethyl) benzene (2.25 g, 1:1 mass:mass). 2,2′-azo-bis-isobutyronitrile (1 mol %) with respect to the total amount of monomers was added. The mixture was placed in a two-necked round bottom flask, equipped with a rubber septum and a valve. The reaction mixture was degassed three times by freeze–pump–thaw cycles and filled back with nitrogen. The reaction was carried out at 65 °C for 40 min. During the reaction time three samples were taken, precipitated in methanol and dried in vacuo at 40 °C until a constant weight was obtained. The conversion was determined gravimetrically. The polymer composition was determined by ^1^H NMR for samples with conversions below 10% after alcoholysis in methanol. The experiment was conducted twice with two different monomer compositions as described in Table 1.

#### 2.3.2. Maleic Anhydride and *n*-Butyl Methacrylate

The copolymerization reactions were performed in analogy to the previously described polymerization of maleic anhydride and 1*H*,1*H*,2*H*,2*H*-perfluorodecyl methacrylate mixtures. The batch compositions are detailed in Table 1.

^1^H NMR (CDCl_3_, δ in ppm) of P [BMA-*co*-MAH]: 3.95 (m, 2H, –O–CH_2_–CH_2_–CH_2_–CH_3_); 3.66 (m, 3H, –O–CH_3_); 2.71 (m, 2H, HOOC–CH–CH–COOMe); 1.93 (m, NN); 1.60 (m, 2H, –O–CH_2_–CH_2_–CH_2_–CH_3_); 1.40 (m, 2H, –O–CH_2_–CH_2_–CH_2_–CH_3_); 0.93 (m, 3H, –O–CH_2_–CH_2_–CH_2_–CH_3_).

#### 2.3.3. 1*H*,1*H*,2*H*,2*H*-Perfluoroodecyl Methacrylate and *n*-Butyl Methacrylate

The copolymerization reactions were performed in analogy to the previously described polymerization of maleic anhydride and 1*H*,1*H*,2*H*,2*H*-perfluorodecyl methacrylate mixtures. The batch compositions are in Table 1.

^1^H NMR (CDCl_3_/Freon-113 (1:1, vol:vol), δ in ppm) P [BMA-*co*-F8H2MA]: 4.34 (m, 2H, –O–CH_2_–CH_2_–(CF_2_)_8_–F); 4.03 (m, 2H, –O–CH_2_–CH_2_–CH_2_–CH_3_); 2.52 (s, 2H, –O–CH_2_–CH_2_–(CF_2_)_8_–F); 1.92 (s, 2H, –C–CH_2_–C– (BMA backbone) overlapped with NN); 1.66 (m, 2H, –O–CH_2_–CH_2_–CH_2_–CH_3_); 1.47 (m, 2H, –O–CH_2_–CH_2_–CH_2_–CH_3_); 1.00 (m, 3H, –O–CH_2_–CH_2_–CH_2_–CH_3_).

#### 2.3.4. 1*H*,1*H*,2*H*,2*H*-Perfluorodecyl Methacrylate, *n*-Butyl Methacrylate and Maleic Anhydride

The terpolymerization reactions were performed in analogy to the previously described polymerization of maleic anhydride and 1*H*,1*H*,2*H*,2*H*-perfluorodecyl methacrylate mixtures. The batch compositions are detailed in Table 1.

^1^H NMR (acetone-*d*_6_/Freon-113 (1:1, vol:vol), δ in ppm): 4.34 (m, 2H, –O–CH_2_–CH_2_–CF_2_–); 4.00 (m, 2H, –O–CH_2_–CH_2_–CH_2_–CH_3_); 3.60 (m, 3H, –COO–CH_3_); 2.8 (s, Freon-113); 1.86 (m, 2H, –O–CH_2_–CH_2_–CF_2_–); 1.65 (m, 2H, –O–CH_2_–CH_2_–CH_2_–CH_3_); 1.45 (m, 2H, –O–CH_2_–CH_2_–CH_2_–CH_3_); 0.98 (m, 3H, –O–CH_2_–CH_2_–CH_2_–CH_3_). Signals in the range of 1–2 ppm are overlapped with the signals of backbone protons.

#### 2.3.5. 1*H*,1*H*,2*H*,2*H*-Perfluoroodecyl Methacrylate, Dodecyl Methacrylate and Maleic Anhydride

The terpolymerization reactions were performed in analogy to the previously described polymerization of maleic anhydride and 1*H*,1*H*,2*H*,2*H*-perfluorodecyl methacrylate mixtures. The batch compositions are detailed in Table 1.

^1^H NMR (CDCl_3_, δ in ppm): 4.19 (m, 2H, –O–CH_2_–CH_2_–CF_2_–); 3.86 (m, 2H, –O–CH_2_–CH_2_–(CH_2_)_9_–CH_3_); 3.60 (m, 3H, –COO–CH_3_); 2.8 (s, Freon-113); 1.82 (m, 2H, –O–CH_2_–CH_2_–CF_2_–); 1.54 (m, 2H, –O–CH_2_–CH_2_–(CH_2_)_9_–CH_3_); 1.19 (m, 2H, –O–CH_2_–CH_2_–(CH_2_)_9_–CH_3_); 0.81 (m, 3H, –O–CH_2_–CH_2_–(CH_2_)_9_–CH_3_). Signals in the range of 1–2 ppm overlapped with the signals of backbone protons.

### 2.4. Continuous Addition Terpolymerization

For details on calculations, please see the Supplementary Materials “Theory of continuous addition polymerization”.

#### 2.4.1. 1*H*,1*H*,2*H*,2*H*-Perfluorodecyl Methacrylate, *n*-Butyl Methacrylate and Maleic Anhydride (10-Fold Monomer Excess)

Preparation of the stock solution (Solution A): *n*-butyl methacrylate (0.448 g, 3.15 mmol), 1*H*,1*H*,2*H*,2*H*-perfluorodecyl methacrylate (0.722 g, 1.45 mmol), maleic anhydride (1.33 g, 13.57 mmol) and 2,2′-azo-bis-isobutyronitrile (0.03 g, 0.18 mmol) were dissolved in a mixture of 2-butanon (4.53 g, 3.63 mL) and 1,3-bis (trifluoromethyl) benzene (2.79 g, 3.63 mL). The mixture was placed in a three neck round bottom flask, equipped with a nitrogen inlet, reflux condenser bearing an oil bubbler vent as nitrogen outlet, a magnetic stirring bar and a rubber septum. The reaction mixture was degassed three times by freeze–pump–thaw cycles and filled back with nitrogen.

Preparation of the monomers feed mixture (Solution B): *n*-butyl methacrylate (11.0 g, 77.46 mmol), 1*H*,1*H*,2*H*,2*H*-perfluorodecyl methacrylate (12.4 g, 23.3 mmol) and maleic anhydride (1.6 g, 16.33 mmol) were dissolved in a mixture of 2-butanon (2.0 g, 2.5 mL) and 3-bis (trifluoromethyl) benzene (2.0 g, 1.45 mL). In a two neck 100 mL round bottom flask equipped with a rubber septum and valve the solution was degassed three times by freeze–pump–thaw cycles and filled back with nitrogen. Subsequently the mixture was transferred into a 50 mL syringe and mounted on a perfusion pump.

Preparation of the initiator feed solution (Solution C): 2,2′-azo-bis-isobutyronitrile (0.066 g, 0,4 mmol) was dissolved in mixture of 2-butanon and 1,3-bis (trifluoromethyl)benzene (1.0 g 1:1 vol:vol). The mixture was degassed in a 10 mL round bottom flask equipped with rubber septum by three freeze–pump–thaw cycles and filled back with nitrogen. The mixture was transferred into a 1 mL syringe and mounted on a perfusion pump.

Continuous addition terpolymerization: The stock solution A was placed in an oil bath pre-heated to 65 °C and after 5 min the continuous addition of solution B and C was started. Solution B was added at a rate of 0.755 mL/h, solution C with a rate of 0.0205 mL/h by means of separate syringe pumps over a period of 2000 min (33 h 20 min) (For details on the addition rate, please see the Supplementary Materials).

After the addition period, the reaction was heated for another 7 h to complete the reaction (post-addition phase). The reaction mixture was cooled to ambient temperature and the product was precipitated in methanol (500 mL). Yield of terpolymer: 23.9 g, 87%; *M*_n_ = 50,500 g/mol, *M*_w_*/M*_n_ = 2.15 (THF-SEC). ^1^H NMR (acetone-*d*_6_/Freon-113 (1:1, vol:vol), δ in ppm): 4.34 (m, 2H, –O–CH_2_–CH_2_–CF_2_–); 4.00 (m, 2H, –O–CH_2_–CH_2_–CH_2_–CH_3_); 3.60 (m, 3H, –COO–CH_3_); 2.8 (s, Freon-113); 1.86 (m, 2H, –O–CH_2_–CH_2_–CF_2_–); 1.65 (m, 2H, –O–CH_2_–CH_2_–CH_2_–CH_3_); 1.45 (m, 2H, –O–CH_2_–CH_2_–CH_2_–CH_3_); 0.98 (m, 3H, –O–CH_2_–CH_2_–CH_2_–CH_3_). Signals in the range of 1–2 ppm overlapped with the signals of backbone protons. Elemental analysis-calculated: C 48.4; H 5.3; O 15.3; F 31.0 wt %; found: C 48.26; H 5.21; O 15.72; F 30.81 wt %.

Terpolymer composition according to ^1^H NMR was: BMA 69 mol %; F8H2MA 21 mol %; MAH 10 mol %. According to elemental analysis the tercopolymer composition was: BMA 68.7; F8H2MA 21.4; MAH 9.9 mol %.

#### 2.4.2. 1*H*,1*H*,2*H*,2*H*,-Perfluorodecyl Methacrylate, *n*-Butyl Methacrylate and Maleic Anhydride (5-Fold Monomer Excess)

The reaction procedure was analogous to the process detailed for the 10-fold excess reaction. The composition of the required solutions is summarized in Table 2. Solution B was fed at a rate of 0.755 mL/h and Solution C was added at a rate of 0.0205 mL/h over a period of 1000 min (16 h 40 min).

Yield: 10.6 g (85% of theoretical). *M*_n_ = 65000, *M*_w_*/M*_n_ = 1.8 (THF-GPC). ^1^H NMR (acetone-*d*_6_/Freon-113 (1:1, vol:vol) δ in ppm): 4.35 (m, 2H, –O–CH_2_–CH_2_–CF_2_–); 3.99 (m, 2H, –O–CH_2_–CH_2_–CH_2_–CH_3_); 3.60 (m, 3H, –COO–CH_3_); 1.86 (m, 2H, –O–CH_2_–CH_2_–CF_2_–); 2.8 (s, Freon-113); 1.65 (m, 2H, –O–CH_2_–CH_2_–CH_2_–CH_3_); 1.46 (m, 2H, –O–CH_2_–CH_2_–CH_2_–CH_3_); 0.97 (m, 3H, –O–CH_2_–CH_2_–CH_2_–CH_3_). Signals in the range of 1–2 ppm overlapped with the signals of backbone protons. Elemental analysis-calculated: C 48.2; H 5.3; O 15.5; F 31.0 wt %; found: C 48.38; H 5.20; O 16.08; F 30.34 wt %. The terpolymer composition according to ^1^H-NMR was: BMA 67 mol %; F8H2MA 21 mol %; MAH 12 mol %. The composition determined by elemental analysis: BMA 64.8; F8H2MA 20.0; MAH 15.2 mol %.

#### 2.4.3. 1*H*,1*H*,2*H*,2*H*,-Perfluorodecyl Methacrylate, *n*-Dodecyl Methacrylate and Maleic Anhydride (5-Fold Monomer Excess)

The reaction procedure was analogous to the process detailed for the 10-fold excess terpolymerization of *n*-butyl methacrylate, 1*H*,1*H*,2*H*,2*H*-perfluorodecyl methacrylate and maleic anhydride. The composition of the required solutions is summarized in Table 2. Solution B was fed at a rate of 1.764 mL/h and Solution C was added at a rate of 0.0809 mL/h over a period of 500 min (8 h 20 min).

Yield: 11.25 g (90% of theoretical). *M*_n_ = 69000, *M*_w_*/M*_n_ = 1.72. ^1^H NMR (CDCl_3_, δ in ppm): 4.19 (m, 2H, –O–CH_2_–CH_2_–CF_2_–); 3.86 (m, 2H, –O–CH_2_–CH_2_– (CH_2_)_9_–CH_3_); 3.60 (m, 3H, –COO–CH_3_); 2.8 (s, Freon-113); 1.82 (m, 2H, –O–CH_2_–CH_2_–CF_2_–); 1.54 (m, 2H, –O–CH_2_–CH_2_–(CH_2_)_9_–CH_3_); 1.19 (m, 2H, –O–CH_2_–CH_2_–(CH_2_)_9_–CH_3_); 0.81 (m, 3H, –O–CH_2_–CH_2_–(CH_2_)_9_–CH_3_). Signals in the range of 1–2 ppm overlapped with the signals of backbone protons. Elemental analysis-calculated: C 58.3; H 7.5; O 13.5; F 20.7 wt %; found: C 57.14; H 7.28; O 12.1; F 23.48 wt %.

According to ^1^H-NMR the terpolymer composition was: DMA—60 mol %; F8H2MA—17 mol %; MAH-23 mol %. The composition as obtained from elemental analysis data: DMA—57.7; F8H2MA—20.00; MAH—22.3 mol %.

### 2.5. Monomethyl Maleate by Methanolysis of Maleic Anhydride

Maleic anhydride (2 g, 20.4 mmol) was dissolved in methanol (20 mL) and placed in a 50 mL round bottom flask, equipped with reflux condenser. After 2 h, half of the reaction mixture was transferred into 50 mL round bottom flask, the solvent was evaporated under reduced pressure and the sample was analyzed by means of ^1^H NMR. The rest of the reaction mixture was refluxed for another 13 h, dried and subsequently investigated by ^1^H NMR.

^1^H NMR (acetone-*d*_6_, δ in ppm): 6.37 (2H, –CH=CH–); 3.71 (3H, –O–CH_3_).

### 2.6. Methanolysis of MAH Copolymers

Polymer sample (40 mg) was dissolved in either dry chloroform or Freon-113 (5 mL) and dry methanol (3 mL) was added. The mixture was placed in a 25 mL round bottom flask and heated under reflux for 24 h. Subsequently, the product was precipitated in a methanol/diethyl ether 1:2 (vol:vol) mixture (15 mL), filtrated and dried at 40 °C under vacuum for 12 h.

## 3. Results and Discussion

Alfrey and Goldfinger [7] published the mathematic description of the kinetics of a terpolymerization reaction. When three monomers copolymerize, there are nine polymer chain growth reactions possible:

(1) $${\sim M}_{i}{}^{•}+M_{j}\overset{k_{\mathrm{ij}}}{\to }{\sim M}_{j}{}^{•}\;R_{\mathrm{ij}}=k_{\mathrm{ij}}{\;[\sim M}_{i}{}^{•}]\cdot {[M}_{j}]$$

where ~M_i_^·^ is the growing polymer chain with monomer i (i = 1,2,3) as an active chain end, M_j_ is the monomer j (j = 1,2,3), R_ij_ is the reaction rate, k_ij_ is the reaction rate constant, and [x] is the concentration of x in the reaction.

The consumption of the monomers in time is described by three kinetic equations:

(2) $$-\frac{d\left[M_{1}\right]}{\mathrm{dt}}=k_{11}\cdot {[\sim M}_{1}{}^{•}]\cdot {[M}_{1}]+k_{21}\cdot {[\sim M}_{2}{}^{•}]\cdot {[M}_{1}]+k_{31}\cdot {[\sim M}_{3}{}^{•}]\cdot {[M}_{1}]$$

(3) $$-\frac{d\left[M_{2}\right]}{\mathrm{dt}}=k_{12}\cdot {[\sim M}_{1}{}^{•}]\cdot {[M}_{2}]+k_{22}\cdot {[\sim M}_{2}{}^{•}]\cdot {[M}_{2}]+k_{32}\cdot {[\sim M}_{3}{}^{•}]\cdot {[M}_{2}]$$

(4) $$-\frac{d\left[M_{3}\right]}{\mathrm{dt}}=k_{13}\cdot {[\sim M}_{1}{}^{•}]\cdot {[M}_{3}]+k_{23}\cdot {[\sim M}_{2}{}^{•}]\cdot {[M}_{3}]+k_{33}\cdot {[\sim M}_{3}{}^{•}]\cdot {[M}_{3}]$$

where [~M_i_^·^] is the concentration of growing polymer chain with monomer i as an active chain end, [M_i_] is the concentration of monomer i, and k_ij_ is the reaction rate constant of the reaction R_ij_.

Usually by the radical polymerization, only the stationary state will be considered where the radical concentration is constant. In case of ternary copolymerization:

(5) $$k_{12}\cdot {[\sim M}_{1}{}^{•}]\cdot {[M}_{2}]+k_{13}\cdot {[\sim M}_{1}{}^{•}]\cdot {[M}_{3}]=k_{21}\cdot {[\sim M}_{2}{}^{•}]\cdot {[M}_{1}]+k_{31}\cdot {[\sim M}_{3}{}^{•}]\cdot {[M}_{1}]$$

(6) $$k_{21}\cdot {[\sim M}_{2}{}^{•}]\cdot {[M}_{1}]+k_{23}\cdot {[\sim M}_{2}{}^{•}]\cdot {[M}_{3}]=k_{12}\cdot {[\sim M}_{1}{}^{•}]\cdot {[M}_{2}]+k_{32}\cdot {[\sim M}_{3}{}^{•}]\cdot {[M}_{2}]$$

(7) $$k_{31}\cdot {[\sim M}_{3}{}^{•}]\cdot {[M}_{1}]+k_{32}\cdot {[\sim M}_{3}{}^{•}]\cdot {[M}_{2}]=k_{13}\cdot {[\sim M}_{1}{}^{•}]\cdot {[M}_{3}]+k_{23}\cdot {[\sim M}_{2}{}^{•}]\cdot {[M}_{3}]$$

The sequence of repeating units in the terpolymer can be predicted from the knowledge of the terpolymerization equations which require six copolymerization parameters of the three binary co-monomer pairs [7,8]. Based on that model (by inserting Equations (5)–(7) into Equations (2)–(4) and some transformations), the monomer incorporation ratios can be described by following equations (Equations (8)–(10)):

(8) $$\frac{d\left[M_{1}\right]}{d\left[M_{2}\right]}=\frac{\left[M_{1}\right]\cdot \left\{\frac{\left[M_{1}\right]}{r_{21}\cdot r_{31}}+\frac{\left[M_{2}\right]}{r_{21}\cdot r_{32}}+\frac{\left[M_{3}\right]}{r_{23}\cdot r_{31}}\right\}}{\left[M_{2}\right]\cdot \left\{\frac{\left[M_{1}\right]}{r_{21}\cdot r_{31}}+\frac{\left[M_{2}\right]}{r_{21}\cdot r_{32}}+\frac{\left[M_{3}\right]}{r_{23}\cdot r_{31}}\right\}}\cdot \frac{\left\{\left[M_{1}\right]+\frac{\left[M_{2}\right]}{r_{12}}+\frac{\left[M_{3}\right]}{r_{13}}\right\}}{\left\{\frac{\left[M_{1}\right]}{r_{21}}+\left[M_{2}\right]+\frac{\left[M_{3}\right]}{r_{13}}\right\}}$$

(9) $$\frac{d\left[M_{1}\right]}{d\left[M_{3}\right]}=\frac{\left[M_{1}\right]\cdot \left\{\frac{\left[M_{1}\right]}{r_{21}\cdot r_{31}}+\frac{\left[M_{2}\right]}{r_{21}\cdot r_{32}}+\frac{\left[M_{3}\right]}{r_{23}\cdot r_{31}}\right\}}{\left[M_{3}\right]\cdot \left\{\frac{\left[M_{1}\right]}{r_{13}\cdot r_{21}}+\frac{\left[M_{2}\right]}{r_{12}\cdot r_{23}}+\frac{\left[M_{3}\right]}{r_{13}\cdot r_{32}}\right\}}\cdot \frac{\left\{\left[M_{1}\right]+\frac{\left[M_{2}\right]}{r_{12}}+\frac{\left[M_{3}\right]}{r_{13}}\right\}}{\left\{\frac{\left[M_{1}\right]}{r_{31}}+\frac{\left[M_{2}\right]}{r_{32}}+\left[M_{3}\right]\right\}}$$

(10) $$\frac{d\left[M_{2}\right]}{d\left[M_{3}\right]}=\frac{\left[M_{2}\right]\cdot \left\{\frac{\left[M_{1}\right]}{r_{21}\cdot r_{31}}+\frac{\left[M_{2}\right]}{r_{21}\cdot r_{32}}+\frac{\left[M_{3}\right]}{r_{23}\cdot r_{32}}\right\}}{\left[M_{3}\right]\cdot \left\{\frac{\left[M_{1}\right]}{r_{13}\cdot r_{21}}+\frac{\left[M_{2}\right]}{r_{12}\cdot r_{23}}+\frac{\left[M_{3}\right]}{r_{13}\cdot r_{23}}\right\}}\cdot \frac{\left\{\frac{\left[M_{1}\right]}{r_{21}}+\left[M_{2}\right]+\frac{\left[M_{3}\right]}{r_{23}}\right\}}{\left\{\frac{\left[M_{1}\right]}{r_{31}}+\frac{\left[M_{2}\right]}{r_{32}}+\left[M_{3}\right]\right\}}$$

where [M_i_] is the concentration of monomer i, and r_ij_/r_ji_ is the copolymerization parameter of monomer pair i/j.

One can fully predict the behavior of the terpolymerization of three monomers if the behavior of the three monomers has been determined pairwise. Unfortunately, above presented equations are not valid for all monomer systems.

In the case that one of the monomers—for example, maleic anhydride (M_3_)—cannot be homopolymerized, two of the copolymerization parameters become zero and a modified equation that contains five copolymerization parameters can be used [9]. The parameter set consists of the “binary” parameters r_12_, r_21_, r_13_ and r_23_ that are experimentally accessible from binary copolymerization experiments with the three monomer pairs, as well as the parameter ρ that must be determined from the evaluation of a terpolymerization experiments (Equations (11)–(14)).

(11) $$\frac{d\left[M_{1}\right]}{d\left[M_{2}\right]}=\frac{\left[M_{1}\right]\cdot \left\{\rho \cdot \frac{\left[M_{1}\right]}{r_{21}}+\frac{\left[M_{2}\right]}{r_{21}}+\rho \cdot \frac{\left[M_{3}\right]}{r_{23}}\right\}}{\left[M_{2}\right]\cdot \left\{\rho \cdot \frac{\left[M_{1}\right]}{r_{12}}+\frac{\left[M_{2}\right]}{r_{12}}+\frac{\left[M_{3}\right]}{r_{13}}\right\}}\cdot \frac{\left\{\left[M_{1}\right]+\frac{\left[M_{2}\right]}{r_{12}}+\frac{\left[M_{3}\right]}{r_{13}}\right\}}{\left\{\frac{\left[M_{1}\right]}{r_{21}}+\left[M_{2}\right]+\frac{\left[M_{3}\right]}{r_{13}}\right\}}$$

(12) $$\frac{d\left[M_{1}\right]}{d\left[M_{3}\right]}=\frac{\left[M_{1}\right]\cdot \left\{\rho \cdot \frac{\left[M_{1}\right]}{r_{21}}+\frac{\left[M_{2}\right]}{r_{21}}+\rho \cdot \frac{\left[M_{3}\right]}{r_{23}}\right\}}{\left[M_{3}\right]\cdot \left\{\frac{\left[M_{1}\right]}{r_{13}\cdot r_{21}}+\frac{\left[M_{2}\right]}{r_{12}\cdot r_{23}}+\frac{\left[M_{3}\right]}{r_{13}\cdot r_{23}}\right\}}\cdot \frac{\left\{\left[M_{1}\right]+\frac{\left[M_{2}\right]}{r_{12}}+\frac{\left[M_{3}\right]}{r_{13}}\right\}}{\left\{\rho \cdot \left[M_{1}\right]+\left[M_{2}\right]\right\}}$$

(13) $$\frac{d\left[M_{2}\right]}{d\left[M_{3}\right]}=\frac{\left[M_{2}\right]\cdot \left\{\rho \cdot \frac{\left[M_{1}\right]}{r_{12}}+\frac{\left[M_{2}\right]}{r_{12}}+\frac{\left[M_{3}\right]}{r_{13}}\right\}}{\left[M_{3}\right]\cdot \left\{\frac{\left[M_{1}\right]}{r_{13}\cdot r_{21}}+\frac{\left[M_{2}\right]}{r_{12}\cdot r_{23}}+\frac{\left[M_{3}\right]}{r_{13}\cdot r_{23}}\right\}}\cdot \frac{\left\{\frac{\left[M_{1}\right]}{r_{21}}+\left[M_{2}\right]+\frac{\left[M_{3}\right]}{r_{23}}\right\}}{\left\{\rho \cdot \left[M_{1}\right]+\left[M_{2}\right]\right\}}$$

(14) $$\rho =\frac{k_{31}}{k_{32}}$$

where [M_i_] is the concentration of monomer i, r_ij_/r_ji_ is the copolymerization parameter of monomer pair i/j, ρ is the terpolymerization parameter, M_1_ is R_H_MA, M_2_ is R_F_MA, and M_3_ is MAH.

The ρ parameter, as all the copolymerization parameters, must be a positive number and must be valid for full range of ternary monomer compositions. The simplest way to determine the ρ parameter is to perform a terpolymerization reaction at the equal monomer fractions ([M_1_] = [M_2_] = [M_3_]). If one determines the composition of such a copolymer, the following is valid:

(15) $$\rho =\frac{b\cdot e-q_{12}\cdot d}{q_{12}\cdot c-a\cdot e}$$

where $a=\frac{1}{r_{12}}+\frac{1}{r_{23}}$, $b=\frac{1}{r_{21}}$, $c=\frac{1}{r_{12}}$, $d=\frac{1}{r_{12}}+\frac{1}{r_{13}}$, $e=\frac{1+b}{1+a}$, and $q_{12}=\frac{d[M_{1}]}{d[M_{2}]}=\frac{F_{1}}{F_{2}}$, and F_i_ is the fraction of monomer i incorporated in the copolymer.

**Scheme 1:**
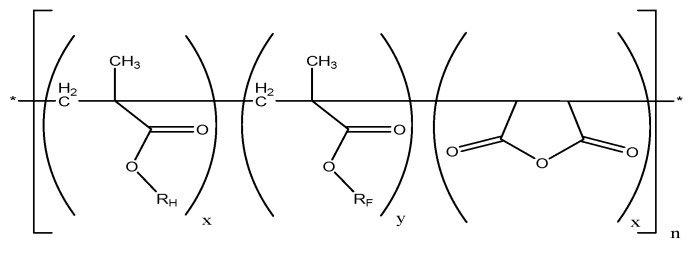
Structure of the target P[R_H_MA-*co*-R_F_MA-*co*-MAH]_n_ terpolymers (x + y + z = 1, R_H_ = C_4_H_9_–, C_12_H_25_–, R_F_– = C_10_H_4_F_19_–).

Terpolymers with succinic anhydride, alkyl (R_H_) methacrylate and perfluoroalkyl (R_F_) methacrylate repeating units (Scheme 1) were designed to combine the outstanding properties of fluorinated polymers with the adhesion promoting properties of polymers containing anhydride and alkyl side groups. Such macromolecules were prepared by free radical terpolymerization of alkyl methacrylates (M_1_ = RHMA), perfluoroalkyl methacrylate (M_2_ = RFMA) and maleic anhydride (M_3_ = MAH). In this study butyl- (R_H_MA = BMA) and dodecyl methacrylate (R_H_MA = DMA) were used as alkyl methacrylates, while 1*H*,1*H*,2*H*,2*H*-perfluorodecyl methacrylate (R_F_MA = F8H2MA) served as monomer to introduce perfluoroalkyl side chains.

**Scheme 2:**
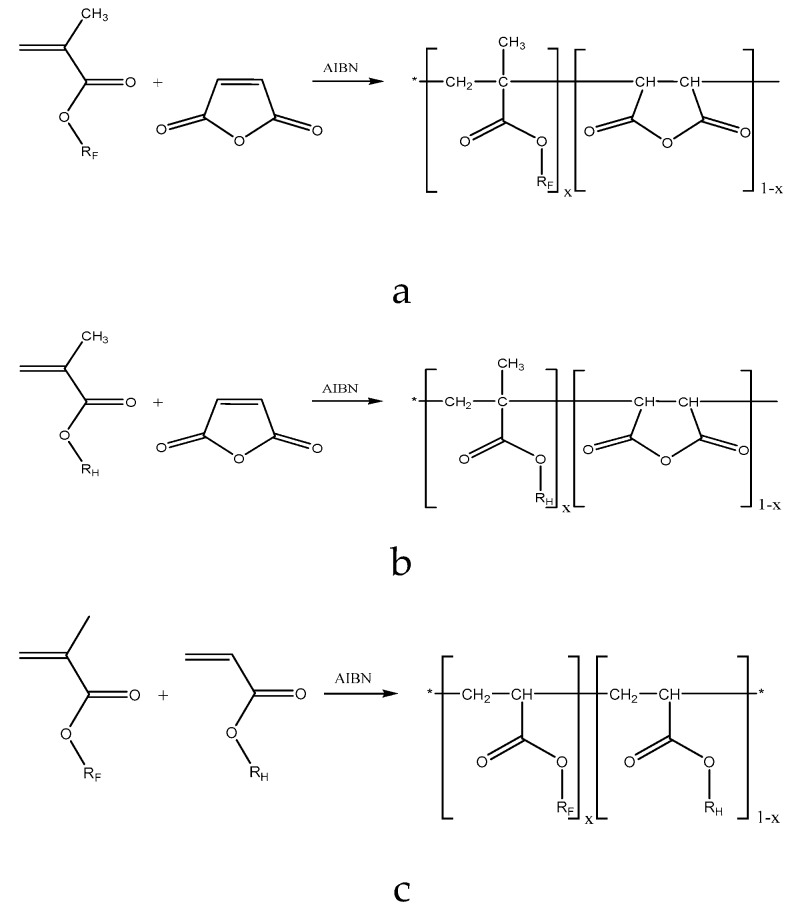
Investigated binary copolymers: (**a**) P[MAH-*co*-F8H2MA]; (**b**) P[MAH-*co*-R_H_MA]; and (**c**) P[R_H_MA-*co*-F8H2MA] with R_H_ = C_4_H_9_– (= BMA), C_12_H_25_– (= DMA) and R_F_– = C_10_H_4_F_19_– (= F8H2MA).

The preparation of well-defined terpolymers from alkyl methacrylates, perfluoroalkyl methacrylates and maleic anhydride (MAH) with uniform microstructure requires the knowledge of copolymerization parameters: the relation between the ratio of monomers in the feed and the ratio of repeating units in the resulting terpolymer as a function of time (Scheme 2).

The respective copolymerization parameters to the best of our knowledge are not known. To obtain the required values, a series of low conversion copolymerization experiments were performed to determine the conversion of the monomers with time. To evaluate the microstructure of the copolymers, the fraction of the monomers incorporated was determined by means of ^1^H NMR spectroscopy at monomer conversions up to 10%. The MAH content of the copolymers could not be determined accurately by measuring the ^1^H NMR spectrum of the crude product; the MAH-proton signals are broad and overlap with signals of the backbone. However, conversion of the anhydride to a monomethyl ester followed by ^1^H NMR analysis and integration of the methyl ester signal an accurate determination of MAH repeating units was possible (for details, see Supplementary Materials).

### 3.1. Low Conversion Copolymerization Experiments with BMA/MAH, F8H2MA/MAH, and F8H2MA/BMA Monomer Pairs, Determination of the Copolymerization Parameters

**Figure 1:**
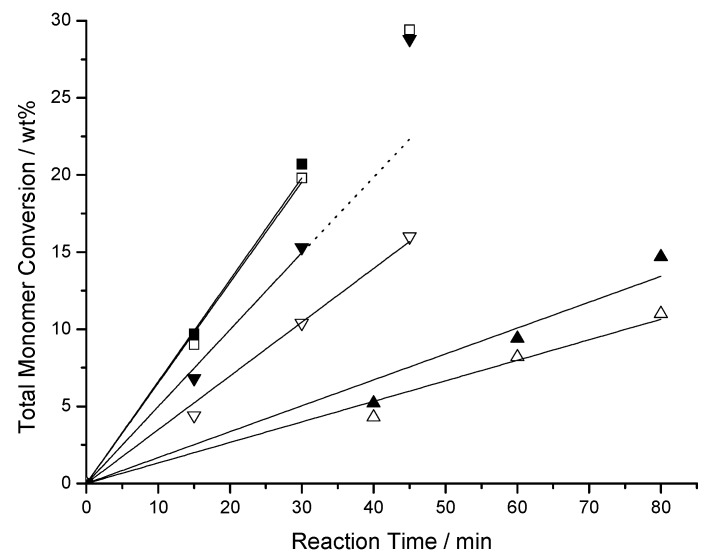
Time conversion plots of binary copolymerization experiments with the comonomer pairs BMA/MAH (■: f_MAH_ = 0.35, ☐: f_MAH_ = 0.60), F8H2MA/MAH (▲: f_MAH_ = 0.3, △: f_MAH_ = 0.6) and F8H2MA/BMA (▼: f_BMA_ = 0.2, ▽: f_BMA_ = 0.45).

The experimental data for the copolymerization of the binary comonomer pairs BMA/MAH, F8H2MA/MAH, and F8H2MA/BMA (for two different monomer compositions) in all cases show a linear increase of the total monomer conversion with time up to conversions of ~12–20%, allowing to determine the initial weight rates of polymerization R_Pw,0_ (Figure 1, Table 3).

The highest rate of polymerization was found with the monomer pair BMA/MAH (f_MAH_ = 0.45: R_Pw,0_ = 0.66 wt %/min), followed by F8H2MA/BMA (f_BMA_ = 0.45: R_Pw,0_ = 0.5 wt %/min) while F8H2MA/MAH was four times slower (f_MAH_ = 0.3: R_Pw,0_ = 0.17 wt %/min). In F8H2MA/MAH mixtures the rate of polymerization decreased with increasing content of MAH in good accordance to prior observations [10]. However, in BMA/MAH mixtures between f_MAH_ = 0.45 and f_MAH_ = 0.6, virtually no change in the rate of polymerization was found. This result is not in agreement with the experimental data for acrylates, but was reported in the literature for copolymerization of MAH with methyl methacrylate [11,12].

**Figure 2:**
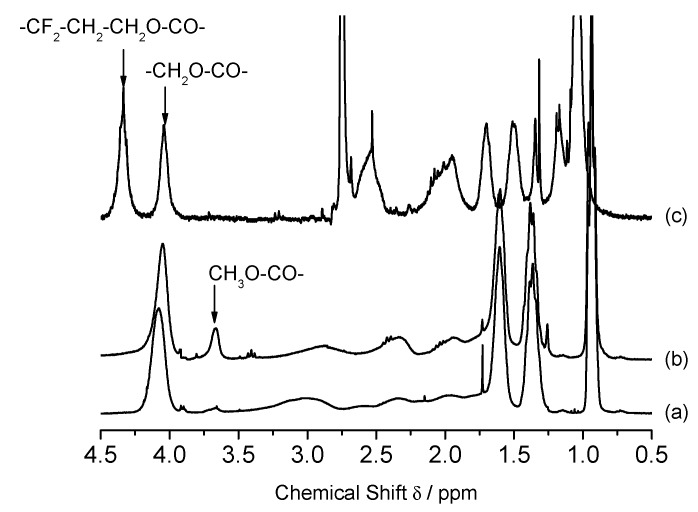
^1^H NMR spectra of: (a) P [BMA_0.9_-*co*-MAH_0.1_]; (b) P[BMA_0.9_-*co*-monomethylmaleate_0.1_] obtained by methanolysis of (a); and (c) P[BMA_0.44_-*co*-F8H2MA_0.56_].

As mentioned, the composition of the copolymers was determined by ^1^H NMR spectroscopy. In Figure 2, typical ^1^H NMR spectra of the binary copolymers are shown. In the spectrum of P[BMA_0.9_-*co*-MAH_0.1_] (Figure 2a), the peak of the butyl ester –COOCH_2_– protons is well resolved at a chemical shift of δ = 4.05 ppm and a very broad signal between 2.7 and 3.5 ppm is caused by one of the succinic anhydride backbone protons. Subsequent to methanolysis (Figure 2b) a new signal appears at δ = 3.7 ppm, caused by the methyl ester group which is well separated from the butyl ester signal. According to Equation (16), 10 mol % MAH units are incorporated into the copolymer. Comparing the spectra of the MAH copolymer and the copolymer after methanolysis it was found that a methyl ester signal of low intensity was already present. Obviously a small fraction (~10%) of the anhydride rings have already been hydrolyzed during the isolation of the polymer by means of precipitation in cold methanol, hence the freshly prepared copolymer is correctly described by the formula P[BMA_0.9_-*co*-MAH_0.09_-*co*-monomethylmaleate_0.01_]. For the further purpose of this work, such a minor degree of methanolysis upon polymer preparation can be tolerated and, in the subsequent text, the copolymers that have not deliberately been boiled with methanol will be treated as pure MAH-copolymers.

(16) $$F_{i}=\frac{A_{i}{/\nu }_{i}}{\sum_{j=1}^{n}A_{j}{/\nu }_{j}}$$

where A_i_ is the integrated intensity of the ^1^H NMR signal of a selected structural element of monomer i, and ν_i_ is the number of protons in the selected structural element of monomer i.

The ^1^H NMR analysis of P[BMA-*co*-F8H2MA] (Figure 2c) does not require any pre-treatment. The signals at 4.34 ppm and 4.03 ppm are assigned to 2H, –O–CH_2_–CH_2_–(CF_2_)_8_–F) and 2H, –O–CH_2_–CH_2_–CH_2_–CH_3_, respectively, are well separated and the composition of the copolymer can easily be calculated.

**Figure 3:**
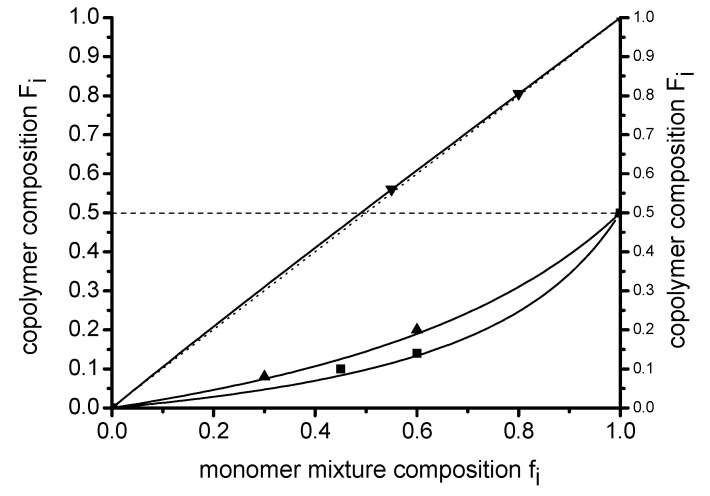
Copolymerization diagrams of the three binary systems F8H2MA/BMA (▼, i = F8H2MA), MAH/F8H2MA (▲, i = MAH) and MAH/BMA (■, i = MAH) at 65 °C.

Figure 3 summarizes the copolymerization diagrams of the three comonomer pairs. The copolymerization line of F8H2MA/BMA is close to an ideal random copolymerization and the nu-merical fit of the integrated Lewis–Mayo equation yielded r_F8H2MA_ = 1.02 and r_BMA_ = 0.94 as most probable values for the copolymerization parameter.

Both MAH copolymer systems exhibit non-ideal copolymerization behavior, characterized by a value of zero for the MAH copolymerization parameter in both cases. Not more than 50 mol % of MAH can hence be incorporated in the copolymers under such circumstances. The obtained copolymerization parameters were r_F8H2MA_ = 4.9, r_MAH_ = 0 and r_BMA_ = 8.2, r_MAH_ = 0. These values are in line with the observed sequences, the total rate of polymerization (R_Pw,0_^F8H2MA/MAH^ > R_Pw,0_^BMA/MAH^) and with other published copolymerization data for MAH/comonomer systems [13].

### 3.2. Terpolymerization Experiments of the BMA/F8H2MA/MAH System

As already mentioned before the obtained results of binary copolymerization of each pair of monomers is not sufficient to describe the behavior of the ternary system in which one of the monomers cannot homopolymerize, hence the necessary data for the ρ-parameter need to be generated by measurements from the ternary monomer mixture. The value of ρ calculated according to Equation (15) is 2.87 (see Supplementary Materials, “Calculation of ρ-parameter”).

To determine the reaction rates of BMA/F8H2MA/MAH mixtures, terpolymerization experiments were performed at three different monomer compositions (Table 4). The composition of the obtained terpolymers was determined by ^1^H NMR spectroscopy. In any case, the monomer conversion was below 15 mol % to ensure a constant monomer composition during the reaction. The reactions were carried out in homogenous solution of HFX:MEK (1:1, vol:vol) with AIBN as the initiator at 65 °C.

**Figure 4:**
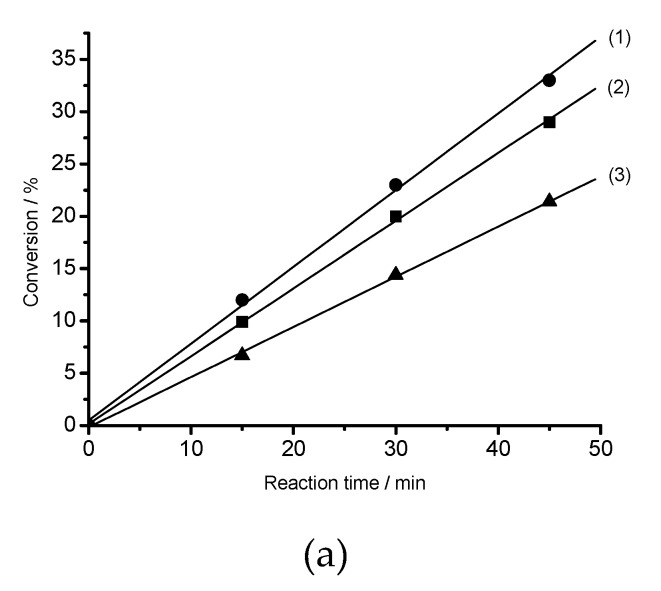

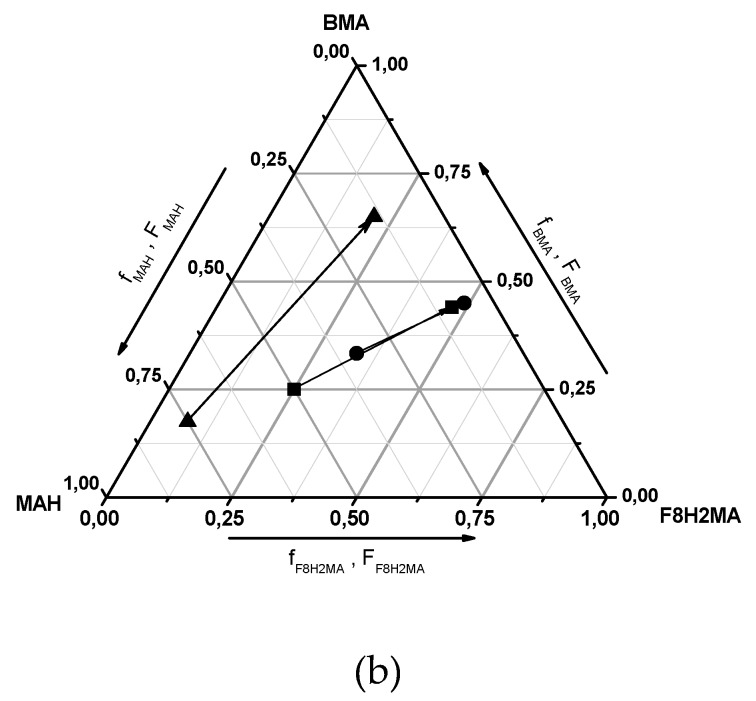
(**a**) Conversion time plots with BMA/F8H2MA/MAH mixtures ((1) ● = 1:1:1, (2) ■ = 1:1:2, (3) ▲ = 1.75:0.75:7.5); and (**b**) the corresponding terpolymerization diagram (the arrows connect monomer and terpolymer composition).

As depicted in Figure 4a, in all cases, the total monomer conversion increased linearly up to conversion of around 20 wt %. In parallel to the binary copolymerization, the presence of MAH reduced the rate of polymerization also in terpolymerization experiments. As for binary copolymers, the presence of maleic anhydride results in a copolymer composition that is different from the composition of monomers in the feed. The differences are depicted in Figure 4b. The arrows in the diagram show the change of the terpolymer composition against the monomer composition in the feed.

### 3.3. Continuous Addition Copolymerization

For the copolymerization of monomers that cannot undergo homopolymerization or that are consumed with different rates, with increasing conversion, the reaction mixture is enriched in the less reactive monomers. The change in the monomer composition with time leads to changing polymer composition. To avoid this phenomenon, the reaction can be stopped at low conversion (usually lower than 10 mol %). In this case the monomer composition is considered to be constant and the variation in copolymer composition is negligible [14].

The huge disadvantage of such a policy is that approximately 90–95% of the monomer mixture will be lost. Hence, this method can only be applied to analytic investigations, such as the determination of copolymerization parameters. For preparative purposes—in particular, for large industrial production—the loss of large quantities of expensive educts cannot be tolerated.

The alternative to low conversion is to deliver the monomers to the reaction mixture with the rate they are consumed. In this way, it is possible to avoid both non-homogenous composition of the desired polymer, and non-reacted monomer mixture as a waste. The theory of continuous addition polymerization with examples has been described in the literature [15] and is summarized in the Supplementary Materials.

To calculate the addition rate, it is necessary to know the copolymerization parameters r_i_ as well as the rate of reaction. When these data are not available in the literature, their determination requires huge effort as demonstrated above. The determination of the r parameters requires series of binary copolymerization and, in the case one of the monomers cannot homopolymerize, additional ternary experiments are necessary to determine the ρ parameter and employ the modified equations. Although the composition of the terpolymer can be calculated, the reaction rate is influenced by many different factors e.g. the type of solvent (presented further on in this text, see Table 8) and need to be determined in a separate ternary copolymerization experiment which is specific for a chosen copolymerization conditions.

Due to the complexity of this procedure, in this work, the following simplified procedure was applied: (i) for a chosen monomer composition, the reaction rate is determined from the conversion vs. time plot; and (ii) the composition of the copolymer is determined for a conversion lower than 10%. (The reactions were performed twice to determine the margin of error. It is of paramount importance to mention that the results obtained are specific for the chosen reaction conditions: monomer composition, monomer and initiator concentration, type of initiator and solvent, temperature etc.) Based on these results, the amounts of monomers, the initiator and the rate of addition are determined. To understand principles of this method we have to define term-“stock solution”. A “stock solution” is a reaction mixture at the time t_0_ = 0, that contains the monomer mixture of the initial concentration C_0_, and the initial mass of monomers m_0_. On the base of these characteristics of the stock solution, the reaction rate and the composition of the copolymer, the amount and rate of addition of monomers and of initiator are calculated. To calculate the reaction time, the so-called “excess α”—meaning the total mass of monomers that has to be added (usually as the multiple of the mass m_0_ in the stock solution)—has to be defined. For continuous terpolymerization with a post addition phase (to complete the conversion of unreacted monomers), 10 times excess (α) (10 × m_0_) has to be added to reduce its influence on the composition of the copolymer.

### 3.4. Continuous Addition Terpolymerization of 1H,1H,2H,2H-Perfluorodecyl Methacrylate (F8H2MA), n-Butyl Methacrylate (BMA) and Maleic Anhydride (MAH) (Copolymers C1 and C2)

**Scheme 3:**
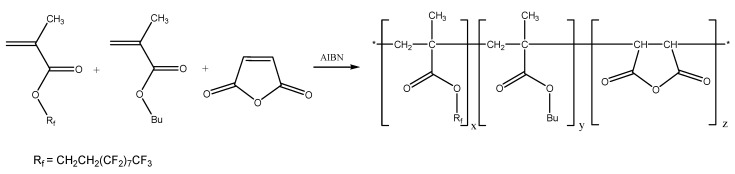
Copolymerization of 1*H*,1*H*,2*H*,2*H*-perfluorodecyl methacrylate with *n*-butyl methacrylate and maleic anhydride (x + y + z = 0.21 + 0.65 + 0.14 = 1).

For the continuous addition experiment the monomer stock solution contained 75 mol % of MAH, 17.5 mol % of BMA and 7.5 mol % of fluorinated monomer (MAH:BMA:F8H2MA = 7.5:1.75:0.75). With such a monomer mixture experiments up to low conversion yielded copolymers (see Scheme 3) with acceptable composition with respect to MAH content and F8H2MA content of 14 and 21 mol % respectively. All parameters necessary to perform the continuous addition experiment were calculated according to the described method (for the details, see Experimental Section and Supplementary Materials).

**Figure 5:**
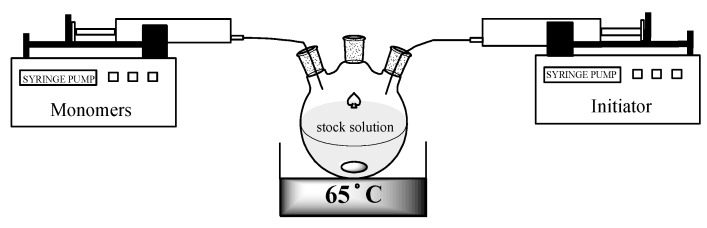
Typical set-up for the continuous addition experiment.

The copolymerization was carried out in a homogeneous mixture of 2-butanone (MEK) with hexafluoroxylene (HFX) 1:1 (vol:vol) and AIBN was used as initiator at 65 °C (Figure 5).

In the first experiment (copolymer C1), a 10-fold excess (α = 10) of monomer with respect to the stock solution was added to decrease the influence of the post addition phase on the homogeneity of the product. The calculated addition time for this experiment was 2000 min (33.3 h). For such a long addition time it is impossible to neglect the change of the initiator concentration in time since at the temperature of the process (65 °C) the half-life time of AIBN is 10 h. For this reason, the initiator had to be continuously added as well (all calculations are available in the Supplementary Materials). To avoid the polymerization of the feed mixture, the solution of the initiator was added separately. Under the reaction conditions even at high monomer conversions no precipitation of the polymer was observed, however the viscosity increased significantly and caused problems with stirring. Such behavior is reasonable for solution containing more than 60 wt % of polymer.

**Figure 6:**
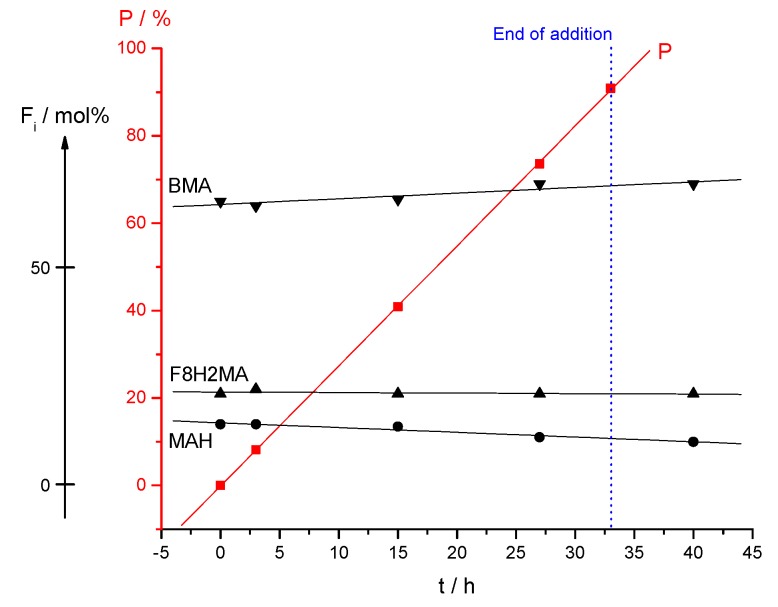
Change of the composition of C1 (P [F8H2MA-*co*-BMA-*co*-MAH]) terpolymer in time during the continuous addition experiment. P represents the conversion plot.

To monitor the progress of the reaction and investigate if the system behaves as initially expected, samples were taken and analyzed during polymerization. The composition of the samples was determined by ^1^H NMR spectroscopy (see Figures S1 and S2) using the method described for low conversion experiments. It was observed that the incorporation of MAH decreased with increasing conversion. Nevertheless, incorporation of fluorinated methacrylate remains constant up to full monomer conversion (see Table 5). The composition drift is depicted in Figure 6. In the case that the low conversion experiment and calculations were done properly, theoretically, no change of the copolymer composition should occur in time.

The low deviation of incorporated monomers (*dF*/*dt*) and its constancy in time seems to be caused by a minimal error in monomer addition rates.

$$\frac{dF_{MAH}}{dt}=\frac{F_{MAH}^{33h}-F_{MAH}^{3h}}{t}=\frac{10-14}{33}\approx -0.12\;\mathrm{mol}\;\%/h;$$

$$\frac{dF_{BMA}}{dt}=\frac{F_{BMA}^{33h}-F_{BMA}^{3h}}{t}\approx +0.13\;\mathrm{mol}\;\%/h;\;\frac{dF_{F8H2MA}}{dt}=\frac{F_{F8H2MA}^{33h}-F_{F8H2MA}^{3h}}{t}\approx -0.03\;\mathrm{mol}\;\%/h$$

Since the measurement of R_p_ is prone to an error of ~10–20%, the achieved accuracy of ~0.1%/h is a reasonable result. The influence of different factors on the possible deviations of terpolymer composition will be discussed later.

The composition of the final products was determined by proton NMR and confirmed by elemental analysis (Table 6).

The slight compositional drift in experiment with 10-fold excess of the monomers could be caused by multiple factors: (i) high viscosity of the reaction mixture and as consequence insufficient stirring; (ii) the so called post-addition phase in which monomers are not supplied to the reaction mixture and the consumption of more reactive monomers; and (iii) incorrect dosage of monomers.

To assess the effect of the two first factors, the experiment with five-fold monomer excess was repeated without post-addition phase (copolymer C2). The composition of the polymer samples taken during the addition period are presented in Table S2. The composition of the final product as a function of the excess α was determined by ^1^H NMR and elemental analysis (Table 7). The amount of incorporated fluorinated methacrylate remains the same while the incorporation of anhydride decreases with the increase of the monomer excess. This clear correlation may be treated as a proof of the reproducibility and robustness of the method but also indicates how important the accuracy of low conversion experiments is.

### 3.5. Continuous Addition Terpolymerization of 1H,1H,2H,2H-Perfluorodecyl Methacrylate (F8H2MA), Dodecyl Methacrylate (DMA) and Maleic Anhydride (MAH) (Copolymer C3)

**Scheme 4:**
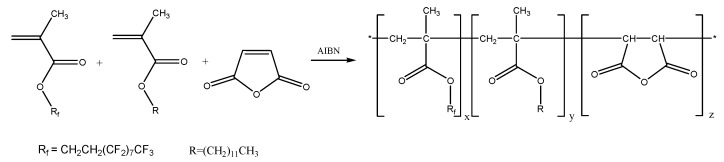
Copolymerization of 1*H*,1*H*,2*H*,2*H*-perfluorodecyl methacrylate with dodecyl methacrylate and maleic anhydride (x + y + z = 0.20 + 0.57 + 0.22 = 1).

To obtain desired properties (e.g., good level of water and oil repellence) of the terpolymer that contains both maleic anhydride and perfluorinated methacrylate, butyl methacrylate was replaced by dodecyl methacrylate (Scheme 4).

This way, one could additionally prove the principles of continuous addition copolymerization working also when other monomers are used. In the first step, low conversion experiments were performed according to the previously described method (see experimental part). Two different monomer compositions were investigated (see Table 8), whereas the initiator concentration was kept constant at 4 mol %.

**Figure 7:**
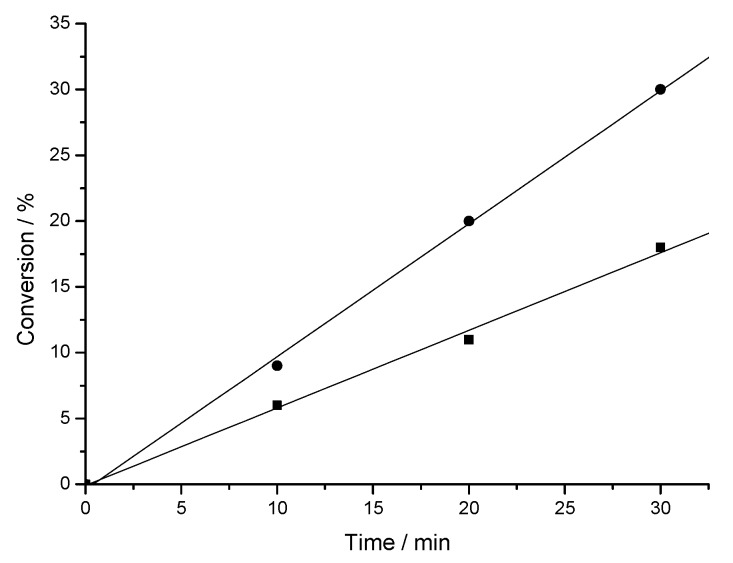
Terpolymerization of DMA, F8H2MA and MAH: time vs. conversion plot for the reaction in MEK (▪) and MEK:HFX 1:1 vol:vol (•).

Because of the low content of the fluorinated monomer in the copolymer the solvent mixture of HFX and MEK was replaced with pure MEK as a solvent to eliminate problematic and expensive fluorinated solvent. The composition of the obtained terpolymer was identical with the one originating from the mixture of solvents. However, the reaction rate was about two times lower in pure MEK than in the mixture of solvents (Figure 7). In conclusion, the composition of the obtained copolymers is identical within the error range for both type of solvents, the reaction rate in pure MEK, however is 40% lower than in MEK/HFX mixture.

For the continuous addition experiment the composition with higher amount of fluorinated monomer was chosen because of higher reaction rate in MEK:HFX 1:1. The determined reaction rate and the composition of copolymer of low conversion experiment were used as base for calculation of the addition rates and composition of the monomer mixture in continuous addition experiment.

**Figure 8:**
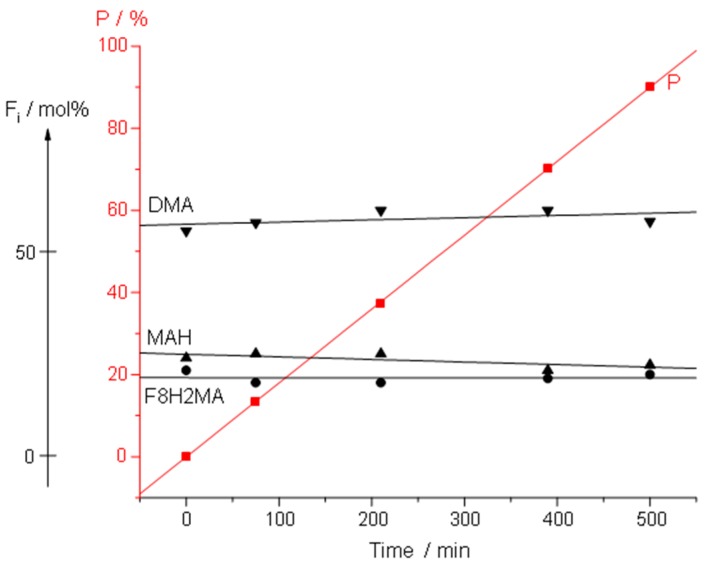
Change of the composition of C3 (P[F8H2MA-*co*-DMA-*co*-MAH]) terpolymer in time in continuous addition experiment. P, conversion; F, copolymer composition; MAH, maleic anhydride; DMA, dodecyl methacrylate; F8H2MA, 1*H*,1*H*,2*H*,2*H*-perfluorodecyl methacrylate.

The feed rates were calculated from theory, based on experimentally measured rates. Due to unavoidable experimental errors, it is hence to be expected that the real feed-rates will slightly deviate from the calculated ones. These mismatches do not play a significant role in short-time experiments, but can become visible in long-time, high conversion reactions. In the present experiment, it was found that certain composition drift occurred during the reaction (Figure 8). Based on it feeding errors have been calculated on the base of NMR data and appear to be higher than in case of BMA copolymer. In addition, the correlation of the composition obtained by NMR spectroscopy and elemental analysis is lower than in the BMA case (see Tables S2 and S3).

It appears that the monomers were misdosed as calculate below:

$$\frac{dF_{MAH}}{dt}=\frac{F_{MAH}^{8h}-F_{MAH}^{0h}}{t}=\frac{24-23}{8.33}\approx -0.2\;\mathrm{mol}\;\%/h;$$

$$\frac{dF_{DMA}}{dt}=\frac{F_{BMA}^{8h}-F_{BMA}^{0h}}{t}\approx +0.3\;\mathrm{mol}\;\%/h;\;\frac{dF_{F8H2MA}}{dt}=\frac{F_{F8H2MA}^{8h}-F_{F8H2MA}^{0h}}{t}\approx -0.1\;\mathrm{mol}\;\%/h$$

Based on the NMR analysis, only the content of DMA has been overestimated; simultaneously, the content of fluorinated methacrylate is higher than determined by magnetic resonance. Comparison between low conversion and continuous addition experiments showed that incorporation of maleic anhydride slowed down slightly during reaction. The copolymer composition calculated from elemental analysis (EA) and ^1^H NMR spectroscopy is presented in Table 9.

Because in the case of low conversion experiment, the composition of polymers was determined only by NMR and has not been checked by elemental analysis, one can assume that compositions of these polymers are comparable to that obtained in continuous addition experiments.

### 3.6. Characterization of the Terpolymers

The molecular weights of synthesized terpolymers were determined by means of GPC in THF as solvent using PMMA calibration standards (Table 10).

In the GPC elugram of the BMA, copolymer a shoulder towards low molecular weight appeared. It is explicitly visible in the elugram of copolymer obtained in 10-fold excess (α = 10) experiment (Figure S5). In this particular case, the viscosity of the reaction mixture became relatively high, and significantly higher than in α = 5 experiment where the GPC shoulder is hardly visible.

In this experiment, as it was assumed, there was no problem with stirring caused by high viscosity, and the GPC elugram also shows almost symmetric distribution (see Figure S5).

Similar effect on the molecular weight distribution has been observed in the case of DMA copolymer. Despite the fact that the experiment was performed with excess of five-fold of monomer, the influence of the viscosity on molecular weight is even more pronounced than in case of BMA (see Figure S6).

The *thermal behavior* of synthesized terpolymers was investigated by means of therogravimetric analysis (TGA) and differential scanning calorimetry (DSC). Thermogravimetic curves of investigated terpolymers show degradation in one step. The thermal stability of the terpolymers is relatively high. A weight loss of 5% has been observed at temperatures of around 300 °C. No significant difference can be noticed with respect to the length of the aliphatic chain in the methacrylate (see Figure S7). The DSC measurements of ternary copolymers (see Figure S8) show only glass transition temperatures and no melting points (Table 11). The single transition is visible, both in the first as well as in the second heating run. The lack of crystallinity in ternary copolymers comparing to binary copolymers with high content of fluorinated methacrylate was also reported by Kraus [16]. The relatively low content of rigid perfluorinated chains which are prone to form ordered structures combined with the presence of “soft” alkyl chains can perfectly explain the experimental data.

An increase of the maleic anhydride content in both n-butyl methacrylate copolymers increases the *T*_g_ value what is in unison with literature data [17,18,19] and is explained by the lower flexibility of the polymer chain. The presence of long alkyl side chains in the copolymer of DMA results in glass transition temperature lower by 20 °C. The lower glass transition is observed despite the fact that relative amount of maleic anhydride and fluorinated moieties compared to alkyl methacrylate is higher than in *n*-butyl methacrylate copolymers. The values of *T*_g_ are collected in Table 11.

## 4. Conclusions

To prepare larger quantities of homogenous terpolymers, the desired composition of the terpolymer can be calculated by means of terpolymerization equations. In the case of terpolymer consisting of butyl methacrylate (BMA), 1*H*,1*H*,2*H*,2*H*-perfluorodecyl methacrylate (F8H2MA) and maleic anhydride (MAH), the copolymerization parameters could not be found in the literature and had to be determined by investigating copolymerization of pairs of monomers. All the experiments were carried out under homogenous conditions in 1:1 (vol:vol) mixture of 2-butanone (MEK) and hexafluoroxylene (HFX) with 2 mol % AIBN as initiator at the temperature of 65 °C. The temperature has been arbitrary chosen as the half-life time of AIBN at this temperature t_1/2_ = 10 h. The total monomer concentration was 2.5 mol/L. The determined copolymerization parameters were: r_F8H2MA_ = 4.9 and r_MAH_ = 0 with the F8H2MA/MAH system; r_BMA_ = 8.2 and r_MAH_ = 0 upon polymerization of BMA/MAH mixtures; and r_F8H2MA_ = 1.02 and r_BMA_ = 0.94 of F8H2MA/BMA.

It has been demonstrated that all the necessary data for continuous addition terpolymerization can be extracted from low conversion ternary experiment but one needs to keep in mind that they are valid only for specific reaction conditions in term of monomer mixture composition, monomer concentrations, concentration and type of radical initiator and reaction temperature. The influence of the solvent on the reaction rate also needs to be considered.

Under the chosen reaction conditions, the rate for monomer mixture 1.75:0.75:7.5 BMA/F8H2MA/MAH was Rp = 0.47 wt %/min and for 1:1:1 BMA/F8H2MA/MAH Rp = 0.73 wt %/min. The composition of the reaction mixture has clear influence on the reaction rate. The determined reaction rates and the composition of the terpolymers were used to perform successfully continuous addition experiments to produce larger quantities of homogenous terpolymers. The versatility of the method has also been proven for a different set of monomers namely dodecyl methacrylate (DMA), 1*H*,1*H*,2*H*,2*H*-perfluorodecyl methacrylate (F8H2MA) and maleic anhydride (MAH). In this case, terpolymer of uniform composition has also been prepared. All the necessary parameters for the continuous addition experiment of DMA/F8H2MA/MAH system were determined in low conversion terpolymerization experiments.

Synthesized copolymers were characterized in terms of molecular weight and thermal properties. DSC measurements of all terpolymers showed glass transition temperatures and absence of melting temperatures. In the case of DMA copolymer, the measured *T*_g_ value was lower than in the case of shorter side alkyl chain (BMA), even though double the amount of anhydride moiety was incorporated into polymer chain.

The developed method of synthesis of large quantity of homogenous terpolymers with relatively low fluorine content opens multiple opportunities for utilization of new coating materials and additives for surface treatment.

## Figures and Tables

**Table 1:** Composition of the reaction mixtures for low conversion copolymerization of monomer systems: MAH/F8H2MA, MAH/BMA, BMA/F8H2MA, BMA/F8H2MA/MAH, DMA/F8H2MA/MAH (*T* = 65 °C).

m_MAH_ (g)	f_MAH_	m_BMA_ (g)	f_BMA_	m_F8H2MA_ (g)	f_F8H2MA_	m_DMA_ (g)	f_DMA_	m_AIBN_ (g)	m_MEK_ (g)	m_HFX_ (g)	C_M_ (mol/L)
0.650	0.60	-	-	2.34	0.400	-	-	0.018	2.25	2.25	2.50
0.220	0.30	-	-	2.77	0.700	-	-	0.013	1.55	1.55	2.50
1.530	0.60	1.400	-	-	-	-	-	0.042	5.15	5.15	2.50
1.080	0.45	1.920	0.400	-	-	-	-	0.040	4.90	4.90	2.50
-	-	0.190	0.550	2.81	0.800	-	-	0.011	1.32	1.32	2.50
-	-	0.540	0.200	2.46	0.550	-	-	0.014	1.70	1.70	2.50
0.360	0.33	0.525	0.450	2.00	0.330	-	-	0.018	2.00	2.00	2.77
0.737	0.50	0.530	0.330	2.00	0.250	-	-	0.025	3.00	3.00	2.50
3.680	0.75	1.240	0.250	2.00	0.075	-	-	0.082	10.00	10.00	2.50
1.840	0.75	-	0.175	1.00	0.075	1.11	0.175	0.164	6.27	6.27	2.00
1.840	0.75	-	-	1.00	0.075	1.11	0.175	0.164	12.54	-	2.00
1.715	0.21	-	-	0.50	0.040	1.25	0.210	0.150	5.84	5.84	2.00
0.920	0.75	-	-	1.00	0.150	0.32	0.100	0.082	3.13	3.13	2.00
2.760	0.90	-	-	0.50	0.030	0.56	0.070	0.200	7.83	7.83	2.00
0.750	0.55	-	-	1.00	0.315	1.11	0.315	0.091	3.48	3.48	2.00

MAH, maleic anhydride; BMA, *n*-butyl methacrylate; F8H2MA, 1*H*,1*H*,2*H*,2*H*-perfluorodecyl methacrylate; DMA, *n*-dodecyl methacrylate;, AIBN, 2,2′-azo-bis-isobutyronitrile; MEK, methyl ethyl ketone; HFX, 1,3-bis (trifluoromethyl) benzene; f_i_, molar fraction of monomer in reaction mixture; C_M_, total monomer concentration.

**Table 2:** Composition of the Stock- and Feed solutions for continuous addition terpolymerization of BMA/F8H2MA/MAH and DMA/F8H2MA/MAH (*T* = 65 °C).

	m_DMA_ (g)	m_BMA_ (g)	m_F8H2MA_ (g)	m_MAH_ (g)	m_AIBN_ (g)	m_MEK_ (g)	m_HFX_ (g)
Stock Solution (A)	-	0.448	0.722	1.33	0.03	4.53	2.79
Monomer Feed Solution (B)	-	5.5	6.2	0.8	---	1.133	1.0
Initiator Feed Solution (C)	-	-	-	-	0.033	0.5	0.5
Stock Solution (A)	0.707	-	0.063	1.16	0.105	3.2	5.51
Monomer Feed Solution (B)	6.497	-	4.698	1.1	-	1.6	2.0
Initiator Feed Solution (C)	-	-	-	-	0.060	0.5	-

BMA, *n*-butyl methacrylate; F8H2MA, 1*H*,1*H*,2*H*,2*H*-perfluorodecyl methacrylate; MAH, maleic anhydride; DMA, *n*-dodecyl methacrylate; AIBN, 2,2′-azo-bisisobutyronitrile; MEK, methyl ethyl ketone; HFX, 1,3-bis (trifluoromethyl) benzene. For details on the calculated amounts, please see the example in Supplementary Materials.

**Table 3:** Monomer mixture in the feed, copolymer compositions and initial weight rate of polymerization of the three monomer pairs MAH/F8H2MA, MAH/BMA and BMA/F8H2MA.

Monomer 1	Monomer 2	f_1_	F_1_	R_Pw,0_/wt % min^−1^
MAH	F8H2MA	0.30	0.080 ± 0.005	0.168 ± 0.008
MAH	F8H2MA	0.60	0.195 ± 0.005	0.133 ± 0.006
MAH	BMA	0.45	0.095 ± 0.005	0.661 ± 0.009
MAH	BMA	0.60	0.140 ± 0.005	0.651 ± 0.008
BMA	F8H2MA	0.20	0.195 ± 0.005	0.589 ± 0.04
BMA	F8H2MA	0.45	0.440 ± 0.01	0.499 ± 0.02

MAH, maleic anhydride; BMA, *n*-butyl methacrylate; F8H2MA, 1*H*,1*H*,2*H*,2*H*-perfluorodecyl methacrylate; R_Pw,0_, initial weight rates of polymerization.

**Table 4:** Composition of monomer mixtures and terpolymers, as well as initial weight rates of polymerizations measured with BMA/F8H2MA/MAH.

	f_BMA_	F_BMA_	f_MAH_	F_MAH_	f_F8H2MA_	F_F8H2MA_	R_p_ (wt %/min)
(1)	0.33	0.45	0.33	*0.06*	0.33	0.49	0.73
(2)	0.25	0.44	0.50	*0.09*	0.25	0.47	0.65
(3)	0.175	0.65	0.75	*0.14*	0.075	0.21	0.47

MAH, maleic anhydride; BMA, *n*-butyl methacrylate; F8H2MA, 1*H*,1*H*,2*H*,2*H*-perfluorodecyl methacrylate; R_p_, initial weight rates of polymerization; f_i_, molar fraction of the monomer in the feed; F_i_, molar fraction of the monomer incorporated into polymer.

**Table 5:** Compositions of the copolymer samples taken during the continuous addition experiment (copolymer C1).

Reaction time (h)	Polymer composition ^1^ (mol %)			
	Conversion (%)	F8H2MA	BMA	MAH
3	8.2	22	64	14
15	40.9	21	65.5	13.5
27	73.6	21	69	11
33 + 7 ^2^	90.9 ^3^	21	69	10
Theoretical composition ^4^		21 ± 2	65 ± 2	14 ± 2

MAH, maleic anhydride; BMA, *n*-butyl methacrylate; F8H2MA, 1*H*,1*H*,2*H*,2*H*-perfluorodecyl methacrylate. ^1^ Determined by ^1^H NMR spectroscopy; ^2^ 7 h after the post-addition phase; ^3^ Conversion calculated for 33 h; ^4^ As determined from low conversion experiment.

**Table 6:** Comparison between the elemental compositions calculated based on magnetic resonance (^1^H-NMR) and measured by elemental analysis (EA).

	Copolymer	Excess α	Carbon (wt %)	Hydrogen (wt %)	Oxygen (wt %)	Fluorine (wt %)
^1^H NMR	C1	10	48.44	5.35	15.31	30.90
EA	C1	10	48.26	5.22	15.71	30.81
^1^H NMR	C2	5	48.19	5.26	15.51	31.04
EA	C2	5	48.38	5.21	16.08	30.33

**Table 7:** Copolymer composition calculated from ^1^H NMR and elemental analysis (EA) at different monomer excess and low conversion experiment (α = 0).

	Copolymer	Excess α	F8H2MA (mol %)	BMA (mol %)	MAH (mol %)
^1^H-NMR	C1	10	21.00	69.00	10.00
EA	C1	10	21.37	68.68	9.95
^1^H-NMR	C2	5	21.00	67.00	12.00
EA	C2	5	20.05	64.77	15.18
^1^H-NMR	-	0 *	21.00	65.00	13.00

MAH, maleic anhydride; BMA, *n*-butyl methacrylate; F8H2MA, 1*H*,1*H*,2*H*,2*H*-perfluorodecyl methacrylate. * Low conversion experiment.

**Table 8:** P(DMA-*co*-F8H2MA-*co*-MAH). Monomer composition in the feed and copolymer composition and the initial reaction rates at low conversion.

Solvent	f_F8H2MA_	f_DMA_	f_MAH_	F_F8H2MA_	F_DMA_	F_MAH_	R_p_ (%/min)
MEK/HFX	0.075	0.175	0.75	0.21	0.55	0.24	1.0
MEK/HFX	0.04	0.21	0.75	0.13	0.62	0.25	0.82
MEK	0.075	0.175	0.75	0.21	0.54	0.25	0.6

MAH, maleic anhydride; DMA, dodecyl methacrylate; F8H2MA, 1*H*,1*H*,2*H*,2*H*-perfluorodecyl methacrylate; MEK, methyl ethyl ketone; HFX, 1,3-bis (trifluoromethyl) benzene; R_P,_ weight rate of polymerization; f_i_, molar fraction of the monomer in the feed; F_i_, molar fraction of the monomer incorporated into polymer.

**Table 9:** Comparison of the copolymer C2 composition calculated from elemental analysis (EA) and ^1^H-NMR data for continuous addition and low conversion (α = 0).

	Excess α	F8H2MA (mol %)	DMA (mol %)	MAH (mol %)
^1^H NMR	5	17.00	60.00	23.00
EA	5	20.00	57.73	22.27
^1^H NMR	0	21.00	55.00	24.00

MAH, maleic anhydride; DMA, dodecyl methacrylate; F8H2MA, 1*H*,1*H*,2*H*,2*H*-perfluorodecyl methacrylate.

**Table 10:** Molecular weights of the copolymers for different value of excess α.

Copolymer	α	*M*n	*M*w	*Đ*
BMA/F8H2MA/MAH	0 *	68,000	108,000	1.59
C1	5	63,000	117,000	1.85
C2	10	50,500	120,000	2.38
DMA/F8H2MA/MAH	0 *	71,500	134,000	1.88
C3	5	69,000	118,000	1.71

BMA, *n*-butyl methacrylate; F8H2MA, 1*H*,1*H*,2*H*,2*H*-perfluorodecyl methacrylate; MAH, maleic anhydride; DMA, dodecyl methacrylate; * α = 0; low conversion experiment; C1 (poly[BMA_0.69_-*co*-F8H2MA_0.21_-*co*-MSA_0.1_]); C2 (poly[BMA_0.67_-co-F8H2MA_0.21_-co-MSA_0.12_]); C3 (poly[DMA_0.57_-*co*-F8H2MA_0.2_-*co*-MSA_0.22_]).

**Table 11:** Composition and glass transition temperatures (second heating) of ternary copolymers obtained in continuous addition experiment. Composition according to elemental analysis.

	F_F8H2MA_	F_Alkyl-MA_	F_MAH_	*T*_g_
C1	21	69	10	56
C2	20	65	15	58
C3	20	58	22	29

MAH, maleic anhydride; Alkyl-MA, *n*-butyl methacrylate (C1 (poly[BMA_0.69_-*co*-F8H2MA_0.21_-*co*-MSA_0.1_]), C2 (poly[BMA_0.67_-*co*-F8H2MA_0.21_-*co*-MSA_0.12_]); dodecyl methacrylate (C3 (poly[DMA_0.57_-*co*-F8H2MA_0.2_-*co*-MSA_0.22_]); F8H2MA, 1*H*,1*H*,2*H*,2*H*-perfluorodecyl methacrylate; F_i_, molar fraction of the monomer incorporated into polymer.

## Supplementary Materials

The supplementary materials are available online at www.mdpi.com/2073-4360/9/11/610/s1.
