# Supplementary material for: Synthesis of Terpolymers with Homogeneous Composition by Free Radical Copolymerization of Maleic Anhydride, Perfluorooctyl and Butyl or Dodecyl Methacrylates: Application of the Continuous Flow Monomer Addition Technique
Source: Polymers (Basel). 2017 Nov 13;9(11):610. doi: 10.3390/polym9110610 (PMC6418581; doi:10.3390/polym9110610)
Supplement: Supplementary file 1 [file polymers-09-00610-s001.pdf]

Synthesis of Terpolymers with Homogeneous Composition by Free Radical Copolymerization of Maleic Anhydride, Perfluorooctyl- and Butyl- or Dodecyl-Methacrylates; Application of the Continuous Flow Monomer Addition Technique.

Marian Szkudlarek[1)], Uwe Beginn[2)], Helmut Keul[1)], Martin Möller[1)]

[1] DWI Leibnitz Institute for Interactive Materials and Institute of Technical and Macromolecular Chemistry, RWTH Aachen University, Forckenbeckstraße 50, D-52056 Aachen, Germany, keul@dwi.rwth-aachen.de

[2] Universität Osnabrück, Institut für Chemie, OMC, Barbarastraße 7, D-49076 Osnabruck, Germany. E-mail: ubeginn@uni-osnabrueck.de

* Correspondence: ubeginn@uni-osnabrueck.de; Tel: +495419692790; Moeller@dwi.rwth-aachen.de; Tel: +492418023302

Determination of MSA units in copolymers:

MSA repeating units were converted in a functionality that allows quantitative determinations. In a model experiment maleic anhydride was reacted with an excess of methanol (Scheme SI1) and the reaction product was analysed by ^{1}H NMR spectroscopy. The ^{1}H NMR spectrum shows signals centred at 6.37 ppm and a singlet at 3.71 ppm. From the coupling pattern and the ratio of the signal intensity (2 : 3, respectively) the product was identified as monomethyl maleate. The absence of a singlet at 6.2 ppm and 7.05 ppm demonstrated the absence of dimethyl maleate.

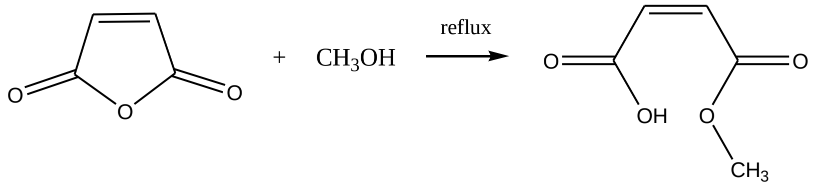

Scheme SI1: Methanolysis of maleic anhydride as model reaction

To measure the maleic anhydride content of a copolymer, the polymer sample was heated in solution in the presence of an excess of methanol (cf. experimental part) to open the anhydride ring in analogy to the reaction depicted in Scheme SI1. Subsequently, the relative MSA content was obtained by comparing the ^{1}H NMR signal intensity of the -CO-OC<u>H</u>$_3$ group at 3.6 – 3.7 ppm to that of the –CO-OC<u>H</u>$_2$- signals of either F8H2MA, BMA or DMA. Table SI1 summarises the chemical shifts and the number of protons per functional group of the four monomer units used in this study. The content of monomer *i* in the copolymer as expressed by its molar fraction F_i has been calculated according to Eq. SI1.

$$F_i = \frac{A_i/\nu_i}{\sum_{j=1}^{n} A_j/\nu_j} \qquad \text{Eq. SI1)}$$

Where:

(A_i = integrated intensity of the ^{1}H NMR signal of a selected structural element of monomer i,
ν_i = number of protons in the selected structural element of monomer i)

Table SI1: Functional groups, ^{1}H NMR chemical shifts and number of protons of these groups used in Eq. SI1 to determine the copolymer compositions (reference signal: $CHCl_3$, 7.26 ppm)

Monomer	Group	ν	σ in ppm (solvent = $CDCl_3$)	σ in ppm (solvent = Freon 113/$CDCl_3$)
F8H2MA	-CO-O-<u>CH2</u>-CH2-CF2-	2	- [1)]	4.20 – 4.40
BMA	-CO-O-<u>CH2</u>-	2	3.90 – 4.10	3.95 – 4.15
DMA	-CO-O-<u>CH2</u>-	2	3.75 – 4.00	3.80 – 4.10
MSA	-CO-O-<u>CH3</u>	3	3.55 – 3.75	3.60 – 3.80

MSA = maleic anhydride, BMA = n-butyl methacrylate, F8H2MA = 1H,1H,2H,2H-perfluorodecyl methacrylate

1) ^{1}H-NMR spectra of F8H2MA copolymers have not been measured in pure $CDCl_3$

The binary copolymerisation parameter r_{ij} and r_{ji} of a monomer pair *i*/*j* were subsequently calculated from a series of copolymer compositions F_i arising from experiments with varying comonomer compositions f_i. The r-values were obtained by numerical fit of the integrated Lewis-Mayo equation (terminal model, Eq. SI2) to the data set by means of the program COPOINT [1].

$$F_i = \frac{r_i \cdot f_i^2 + f_i \cdot f_j}{r_i \cdot f_i^2 + 2 \cdot f_i \cdot f_j + r_j \cdot f_j^2} \qquad \text{Eq. SI2)}$$

(F_i – molar fraction of monomer i in the copolymer, f_i – molar fraction of monomer i in the reaction mixture, $f_j = 1 - f_i$)

Theory of continuous addition polymerization

The change of the monomer fraction f_i with the conversion:

$$\frac{df_i}{dp} = \frac{f_i - F_i}{1-p} \quad \text{for } p \to 0 \qquad \text{Eq. SI3)}$$

(f_i – molar fraction of monomer "*i*" in the reaction mixture; F_i – molar fraction of monomer "*i*" incorporated in polymer; *p* – conversion)

$$\frac{df_i}{dp} \approx f_i - F_i \qquad \text{Eq. SI4)}$$

$$\Delta f_i \approx (f_i - F_i) \cdot \Delta p \qquad \text{Eq. SI5)}$$

for $\Delta p \to 0$

$\Delta f_i \to 0$

it means that if the conversion change Δp is close to 0 also the change of the monomer fraction Δf_i in the mixture is close to 0.

With monomer addition:

$$\frac{df_i}{dp} = \frac{f_i - F_i}{q-p} + \frac{\alpha_i - f_i}{q-p} \cdot \frac{dq}{dp} = 0 \qquad \text{Eq. SI6)}$$

$$q=\frac{n_0+n^{add}}{n_e^{in}} \quad ; \qquad \alpha_i=\frac{dq_i/dp}{dq/dp}$$

(f_i – fraction of monomer *"i"* in the feed; F_i – fraction of monomer *"i"* incorporated in the polymer; p – conversion; α – addition factor (excess); q – number of moles of monomers in the reaction mixture)

To calculate the addition rate it is necessary to know the copolymerisation parameters r_i as well as the rate of reaction. When these data are not available in the literature, their determination requires huge effort.

The advantage of the method described in this chapter consists on following, simple procedure. For the chosen monomer composition copolymerization reaction has been performed:

- the reaction rate has been determined from conversion vs. time plot.
- the composition of the copolymer was determined for conversion lower than 10 %.

The reaction has been performed twice to eliminate the error. It is of paramount importance to mention that determined parameters are specific for chosen conditions: monomer's composition, concentration of monomers and the initiator, type of the initiator, temperature and solvents.

To understand principles of this method we have to define term- "stock solution". A "stock solution" is a reaction mixture at the time t_0=0, that contains initial the monomer mixture of concentration C_0, and the initial mass of monomers m_0. On the base of "parameters" of the stock solution, reaction rate and the composition of the copolymer, one can calculate the amounts of monomers, the initiator and rate of its addition.

Determined reaction rate is equal to the rate of addition:

$$R_p=\frac{dm}{dt} \qquad \text{Eq. SI7)}$$

$$R_p=\sum_{i=1}^{n}R_{pmi} \qquad \text{Eq. SI8)}$$

For ternary system:

$$R_p=R_{pmi}+R_{pmj}+R_{pmk} \qquad \text{Eq. SI9)}$$

for

$$R_{pmi}=\frac{dm_i}{dt} \qquad \text{Eq. SI10)}$$

and

$$m_i=M_i n_i \qquad \text{Eq. SI11)}$$

(m_i – mass of the monomer "i"; M_i – molecular weight of monomer "i"; n_i – number of moles of the monomer "i")

After the transformation:

$$\frac{dm}{dt}=\frac{dm_i}{dt}\cdot\left(1+\frac{M_j}{M_i}\cdot\frac{dn_j}{dn_i}+\frac{M_k}{M_i}\cdot\frac{dn_k}{dn_i}\right) \qquad \text{Eq. SI12)}$$

$$\frac{\frac{dn_j}{dt}}{\frac{dn_i}{dt}}=\frac{F_j}{F_i}=\frac{dn_j}{dn_i} \qquad \text{Eq. SI13)}$$

(F_i, F_j – denote the content of monomer "i" and "j" in the copolymer)

After the transformation of equation SI12 one can calculate the mass of monomer "i" which has to be added in time unit:

$$\frac{dm_i}{dt} = \frac{\frac{dm}{dt}}{1 + \frac{M_j \cdot F_j}{M_i \cdot F_i} + \frac{M_k \cdot F_k}{M_i \cdot F_i}}$$ Eq. SI14)

For other two monomers:

$$\frac{dm_j}{dt} = \frac{F_j \cdot M_j}{F_i \cdot M_i} \cdot \frac{dm_i}{dt}$$ Eq. SI15)

$$\frac{dm_k}{dt} = \frac{F_k \cdot M_k}{F_i \cdot M_i} \cdot \frac{dm_i}{dt}$$ Eq.SI16)

In order to calculate the addition time one need up front to assume so-called "excess α" – it means the total mass of the monomers which has to be added (usually as the multiple of the mass m_0 in the stock solution). For continuous terpolymerization with post addition phase (to complete the conversion of unreacted monomers) 10 times excess (α) (10 x m_0) has to be added to reduce its influence on the composition of the copolymer.

$$t = \frac{\alpha}{\frac{dm}{dt}}$$ Eq. SI17)

t – addition time
α – total mass of the added monomer mixture ("excess")

Multiplying the mass of the monomer which has to be added in time unit $\frac{dm_i}{dt}$ by the addition time t, one obtains the mass of the monomer.

$m_i = \frac{dm_i}{dt} \cdot t$ The sum of calculated monomer mass has to be equal to the excess:

$$\sum m_i = \alpha$$ Eq. SI18)

It is very important to remember that pumps dose volume and do not dose mass. It is necessary to recalculate masses to volumes using densities.

$$V_i = \frac{m_i}{d_i}$$ Eq. SI19)

(V_i – volume of monomer "i"; m_i – mass of monomer "i"; d_i – density of monomer "i")

In case one of the monomers is not soluble in others it is necessary to use solvent. This should be also considered in calculations of the addition rates. Addition rates are calculated as follow:

$$R_{add} = \frac{\sum V_i}{t}$$ Eq. SI20)

where $\sum V_i$ consists of volume of each component of the monomer mixture.

The required amount of the initiator can be calculated:

$$\frac{dm_{ini}}{dt} = k_{ini} \cdot m_{ini0}$$ Eq. SI21)

where:

$$k_{ini} = \frac{\ln 2}{t_{1/2}}$$ Eq. SI22)

(m_{ini0} – initial mass of the initiator in the stock solution; k_{ini} – decomposition rate constant of the initiator; $t_{1/2}$ – half life time of the initiator at given temperature)

<u>Calculation of ρ – parameter:</u>

$$\rho = \frac{b \cdot e - q_{12} \cdot d}{q_{12} \cdot c - a \cdot e}$$

Where: $a = \frac{1}{r_{12}} + \frac{1}{r_{23}}$, $b = \frac{1}{r_{21}}$, $c = \frac{1}{r_{12}}$, $d = \frac{1}{r_{12}} + \frac{1}{r_{13}}$, $e = \frac{1+b}{1+a}$,

$$q_{12} = \frac{d[M_1]}{d[M_2]} = \frac{F_1}{F_2}$$

r_{12}=0.94, r_{21}=1.02, r_{31}=0, r_{13}=4.9, r_{32}=0, r_{23}=8
F_1=0.45, F_2=0.49

$$a = \frac{1}{0.94} + \frac{1}{8.2} = 1.185$$

$$b = \frac{1}{1.02} = 0.980$$

$$c = \frac{1}{0.94} = 1.064$$

$$d = \frac{1}{0.94} + \frac{1}{4.9} = 1.268$$

$$e = \frac{1+0.980}{1+1.185} = 0.906$$

$$q_{12} = \frac{0.45}{0.49} = 0.918$$

$$\rho = 2.87$$

<u>Example of the calculation:</u>

Targeted terpolymer composition:

F_{F8H2MA}= 0.20	M_{F8H2MA}= 532 g/mol
F_{BMA}= 0.66	M_{BMA}= 142 g/mol
F_{MAH}= 0.14	M_{MAH}= 98 g/mol

Reaction rate: R_p= 0.5 %/min

A) Stock solution

m_0= 2.5 g (arbitrary)
C_0= 2,5 mol/L
Stock solution composition:
m_{BMA} = 0,448 g (3.15 x 10^{-3} mol)
m_{F8H2MA}= 0,722 g (1.45 x 10^{-3} mol)
m_{MSA} = 1.33 g (13.57 x 10^{-3} mol)

m_{MEK} = 4,53 g (3.63 mL)
m_{HFX} = 2,79 g (3.63 mL)
m_{AIBN} = 0,03 g (0.18 x 10^{-3} mol)

B) Addition rate

$$\frac{dm}{dt} = m_0 \cdot R_p = 2.5 \cdot 0.005 = 0.0125 \text{ g}$$

$$\frac{dm_{F8H2MA}}{dt} = \frac{\frac{dm}{dt}}{1 + \frac{M_{BMA} \cdot F_{BMA}}{M_{F8H2MA} \cdot F_{F8H2MA}} + \frac{M_{MAH} \cdot F_{MAH}}{M_{F8H2MA} \cdot F_{F8H2MA}}} = \frac{0.0125}{1 + \frac{142 \cdot 0.66}{532 \cdot 0.2} + \frac{98 \cdot 0.14}{532 \cdot 0.2}} = 0.0062 g / \min$$

$$\frac{dm_{BMA}}{dt} = \frac{F_{BMA} \cdot M_{BMA}}{F_{F8H2MA} \cdot M_{F8H2MA}} \cdot \frac{dm_{F8H2MA}}{dt} = \frac{0.66 \cdot 142}{532 \cdot 0.2} \cdot 0.0062 = 0.0055 g / \min$$

$$\frac{dm_{MAH}}{dt} = \frac{F_{MAH} \cdot M_{MAH}}{F_{F8H2MA} \cdot M_{F8H2MA}} \cdot \frac{dm_{F8H2MA}}{dt} = \frac{0.14 \cdot 98}{532 \cdot 0.2} \cdot 0.0062 = 0.0008 g / \min$$

C) Addition time

α=10 (10x m_0 - arbitrary)

$$t = \frac{\alpha}{\frac{dm}{dt}} = \frac{\alpha}{R_p} = \frac{10}{0.0125} = 33.33 h$$

D) Initiator

$t_{1/2}$= 10 h (AIBN at 65 °C)

$$k_{AIBN} = \frac{\ln 2}{t_{1/2}} = 0{,}012 \min^{-1} \quad (0{,}0693 \text{ h}^{-1})$$

$$\frac{dm_{AIBN}}{dt} = k_{AIBN} \cdot m_{AIBN\,0} = 0.0693 \cdot 0.03 = 0.002 g / h$$

$$\Delta m_{AIBN} = t \cdot \frac{dm_{AIBN}}{dt} = 33.33 \cdot 0.002 = 0.066 g$$

E) Densities necessary to calculate volumes

d_{F8H2MA} – 1,596 g/cm^3
d_{BMA} – 0.90 g/cm^3
d_{MAH} – 1.32 g/cm^3
d_{MEK} – 0.80 g/cm^3
d_{HFX} – 1.378 g/cm^3
d_{AIBN} – 1.11 g/cm^3

F) Monomer mixture composition for addition

$$\frac{dm_{F8H2MA}}{dt} = 0.0062 g / \min = 0.372 g / h$$

$$m_{F8H2MA} = \frac{dm_{F8H2MA}}{dt} \cdot t = 0.372 \cdot 33.33 = 12.4g$$

$$V_{F8H2MA} = \frac{m_{F8H2MA}}{d_{F8H2MA}} = \frac{12.4}{1.596} = 7.77cm^3$$

$$\frac{dm_{BMA}}{dt} = 0.0055g/\min = 0.33g/h$$

$$m_{BMA} = \frac{dm_{BMA}}{dt} \cdot t = 0.33 \cdot 33.33 = 11.0g$$

$$V_{BMA} = \frac{m_{BMA}}{d_{BMA}} = \frac{11}{0.9} = 12.22cm^3$$

$$\frac{dm_{MAH}}{dt} = 0.0008g/\min = 0.048g/h$$

$$m_{MAH} = \frac{dm_{MAH}}{dt} \cdot t = 0.048 \cdot 33.33 = 1.6g$$

$$V_{MAH} = \frac{m_{MAH}}{d_{MAH}} = \frac{1.6}{1.32} = 1.21cm^3$$

MAH is not soluble in the methacrylates used, additional solven was used:

$$V_{MEK} = \frac{m_{MEK}}{d_{MEK}} = \frac{2}{0.8} = 2.5cm^3$$

$$V_{HFX} = \frac{m_{HFX}}{d_{HFX}} = \frac{2}{1.387} = 1.45cm^3$$

Total volume to be added: $V_m = 25.15cm^3$

G) Addition rates (volume)

Monomers: $\frac{V_m}{t} = 0.755mL/h$

Initiator: 0.66 g of AIBN in 0.62 mL of MEK (minimum amount to obtain stable solution)

$$V_{AIBN} = 0.685cm^3$$

$$\frac{V_{ini}}{t} = \frac{V_{AIBN} + V_{MEK}}{t} = \frac{0.685 + 0.62}{33.33} = 0.0205mL/h$$

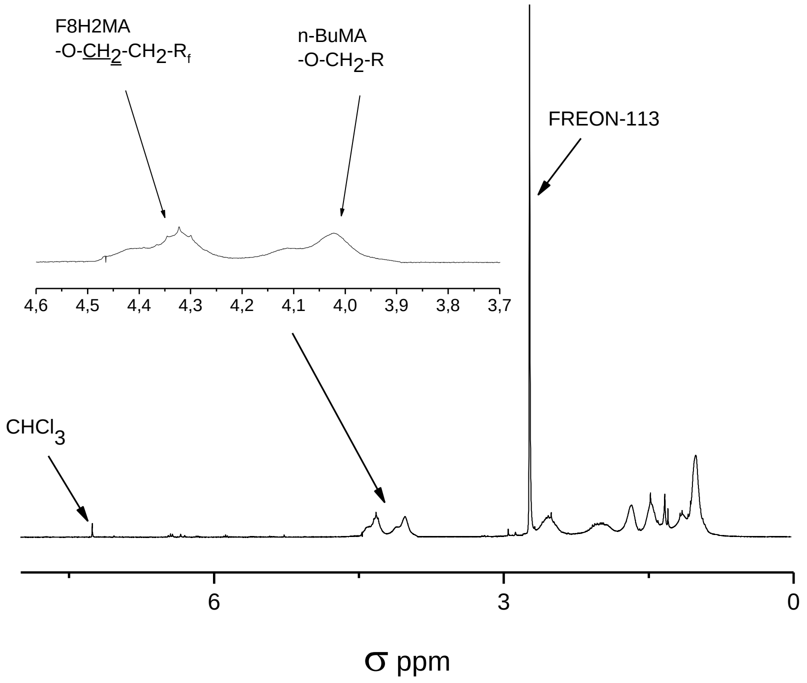

Figure SI 1: ^{1}H-NMR spectra of P[F8H2MA-co-BMA-co-MSA]

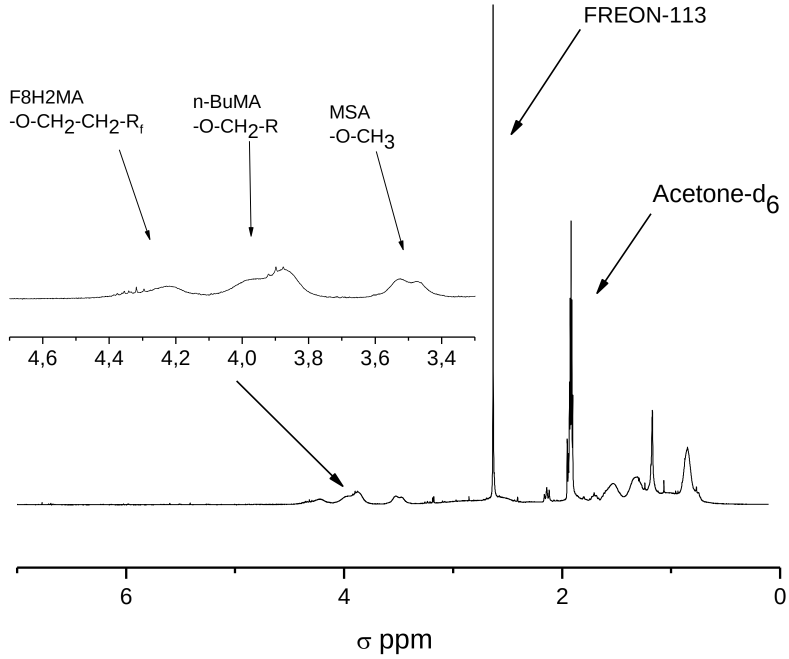

Figure SI2: ^{1}H-NMR spectra of P[F8H2MA-co-BMA-co-MSA] after alcoholysis of succinic anhydride ring with methanol. The spectra were measured in acetone-d_6 as co solvent because of relatively high polarity of hydrolysed copolymer.

The continuous addition polymerization reaction was carried out under homogenous conditions with AIBN as initiator. During the reaction samples were taken and their composition was determined by means of ^{1}H-NMR spectroscopy. In order to avoid problems with stirring and influence of post-addition phase on composition as well as on the molecular weight distribution a 5-fold excess of

monomers was used and reaction was interrupted after the addition phase. The product was isolated by precipitation in methanol and dried in vacuo at 40 ºC to the constant weight. The ^{1}H-NMR of the polymer is depicted in Figure SI3.

In order to determine the MSA content the sample was refluxed in the presence of methanol. The signal from -CO-OC<u>H</u>$_3$ group at 3.6- ppm originated from the metahnolized succinic anhydride ring incorporated in the polymer (see Figure SI4) was used to calculate the copolymer composition.

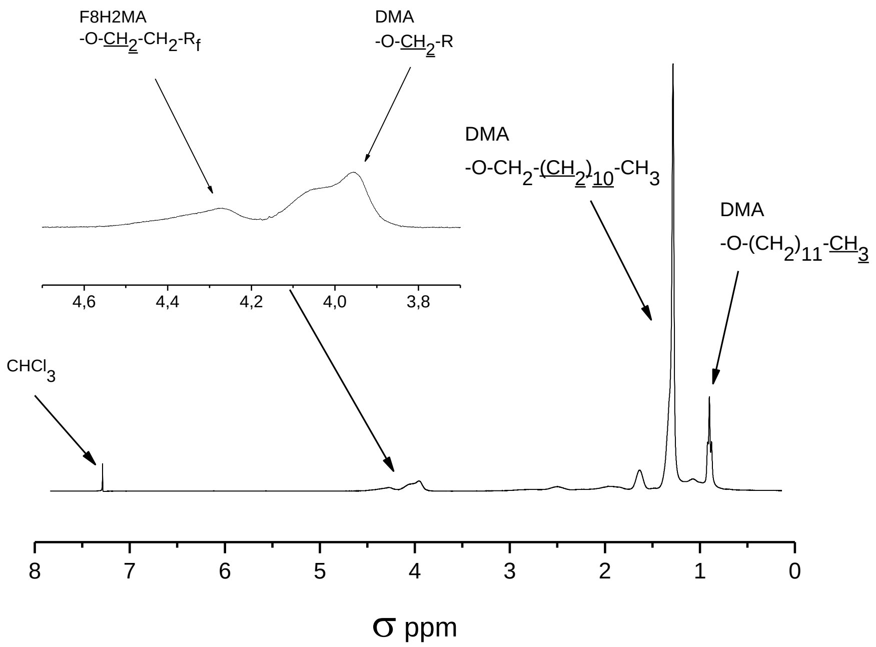

Figure SI3: ^{1}H-NMR spectra of poly[F8H2MA-DMA-MSA] before alcoholysis in methanol.

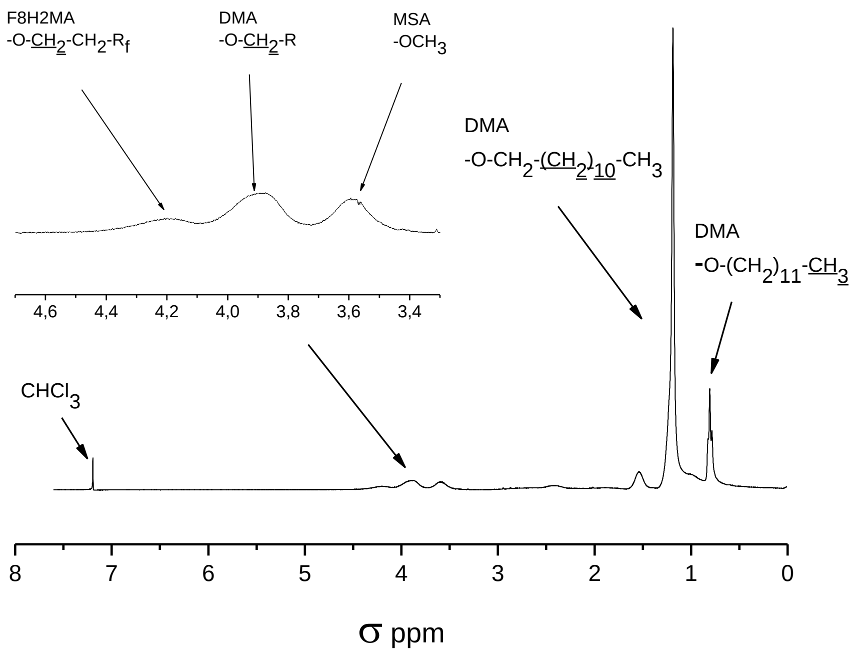

Figure SI4: ^{1}H-NMR spectra of poly[F8H2MA-DMA-MSA] (final product) after alcoholysis in methanol.

Table SI2: Changes in the composition of the copolymer during the continuous addition experiment determined by ^{1}H-NMR.

Reaction time [min]	Polymer composition [1)] [mol %]			
	**Conversion [%]**	**F8H2MA**	**DMA**	**MSA**
75	13.5	18	57	25
210	37.4	18	57	25
390	70.2	19	60	21
500	90	17	60	23
Theoretical composition [2)]		21±2	55±2	24±2

MSA = maleic anhydride, DMA = dodecyl methacrylate, F8H2MA = 1H,1H,2H,2H-perfluorodecyl methacrylate

[1)] Determined by ^{1}H-NMR on sample taken after the indicated reaction time

[2)] As determined from low conversion experiment

Table SI3: Comparison between the elemental compositions calculated on the basis of proton magnetic resonance and measured by elemental analysis (EA).

	**Carbon [wt%]**	**Hydrogen [wt%]**	**Oxygen [wt%]**	**Fluorine [wt%]**
^{1}H-NMR	58.33	7.53	13.44	20.70
EA	57.14	7.29	12.09	23.48

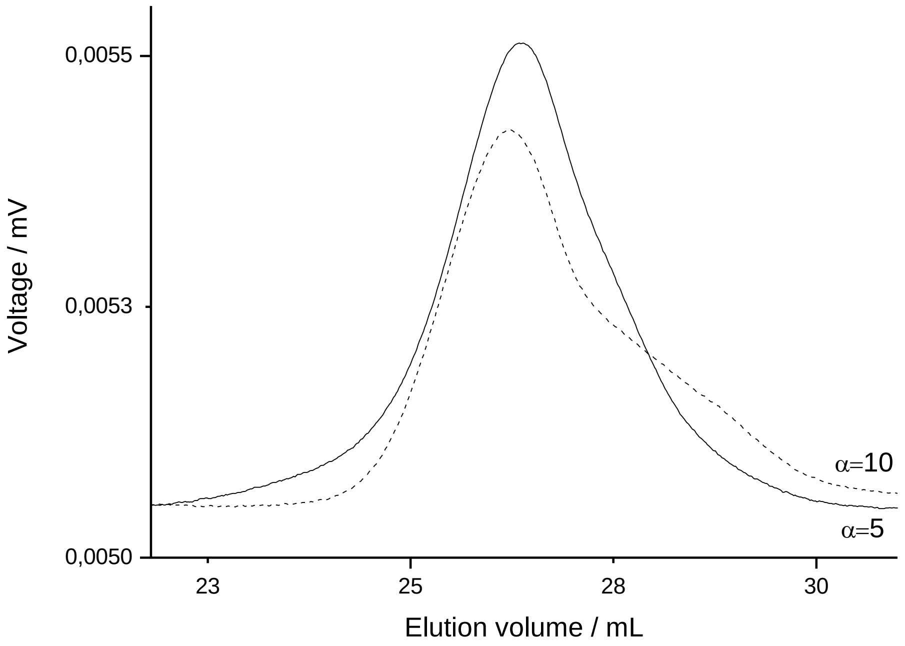

Figure SI5: Molecular weight distribution of the BMA copolymers obtained in continuous addition experiment with the monomer excess α=5 (___) and α=10 (---)

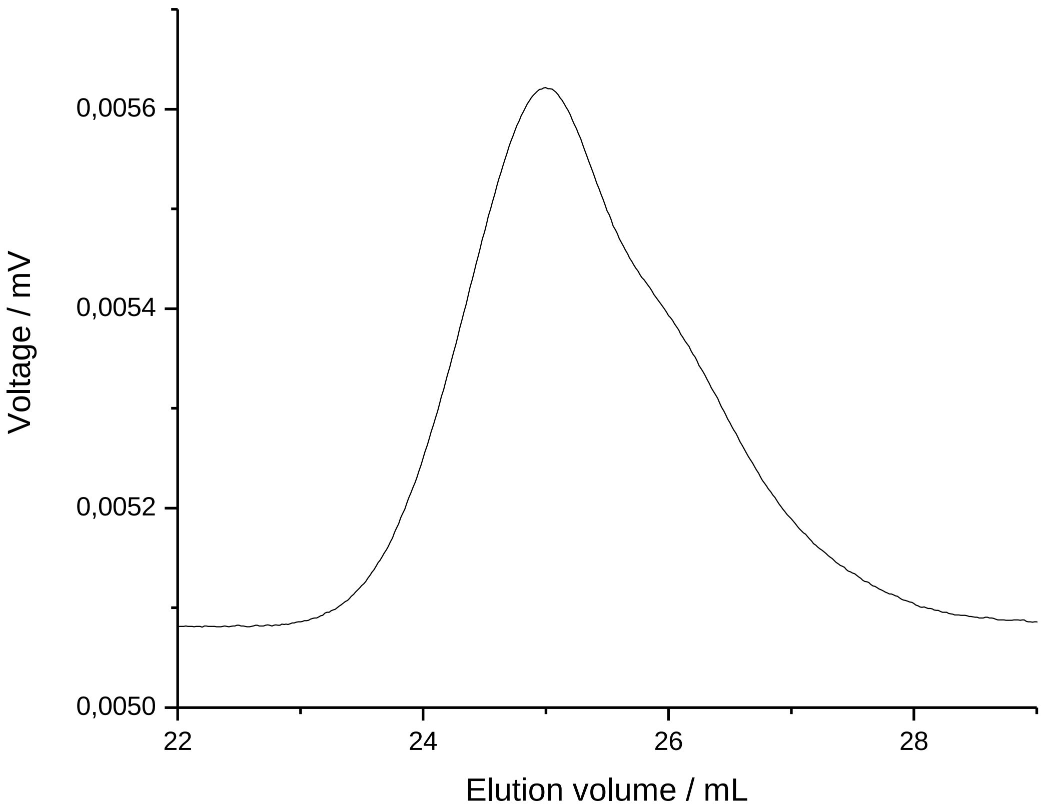

Figure SI6: Molecular weight distribution of poly[DMA-co-F8H2MA-co-MSA] obtained in continuous addition experiment (α=5).

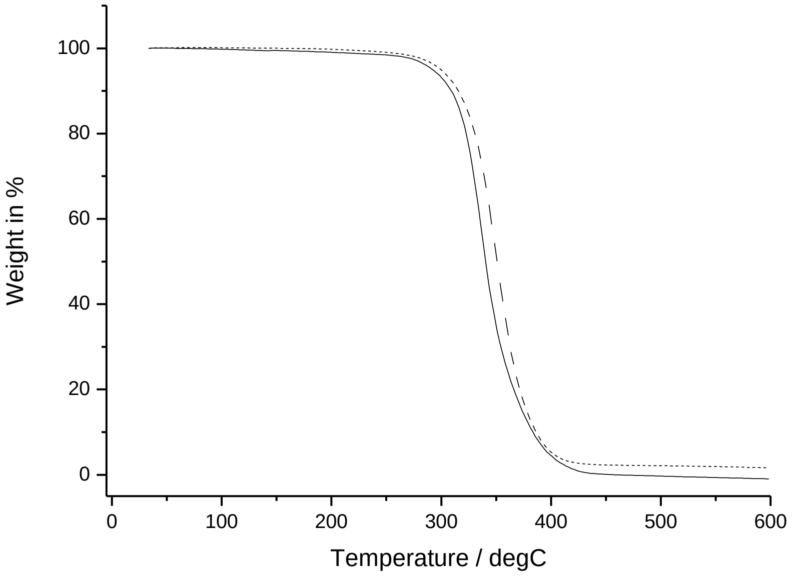

Figure SI7: TGA measurement of fluorinated ternary copolymers under nitrogen. (___) C1 (poly[$BMA_{0.69}$-co-$F8H2MA_{0.21}$-co-$MSA_{0.1}$]), (---) C3 (poly[$DMA_{0.57}$-co-$F8H2MA_{0.2}$-co-$MSA_{0.22}$]).

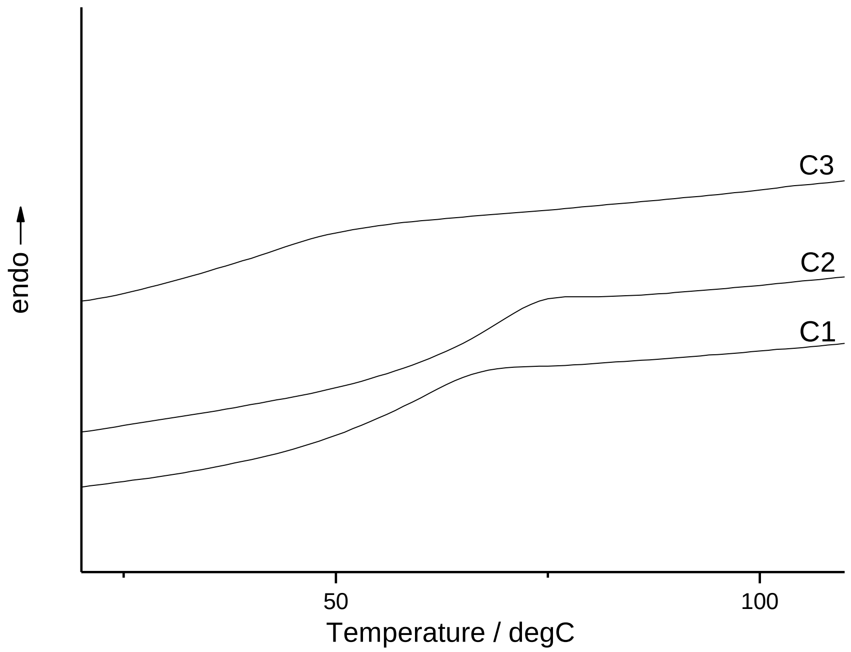

Figure SI 8: DSC thermogram of C1 (poly[$BMA_{0.69}$-co-$F8H2MA_{0.21}$-co-$MSA_{0.1}$]) and C2 (poly[$BMA_{0.67}$-co-$F8H2MA_{0.21}$-co-$MSA_{0.12}$]) C3 (poly[$DMA_{0.57}$-co-$F8H2MA_{0.2}$-co-$MSA_{0.22}$]). The second heating at 10 K/min.

References:

[1] U. Beginn, e-Polymers; 2005; no. 073
